# An Approach Toward Radioiodination and Radiopharmacological Evaluation of a Carborane-Containing Analog of Indomethacin

**DOI:** 10.3390/molecules31111944

**Published:** 2026-06-03

**Authors:** Jonas Schädlich, Christoph Selg, Cathleen Haase-Kohn, Martin Ullrich, Robert Wodtke, Klaus Kopka, Evamarie Hey-Hawkins, Jens Pietzsch, Markus Laube

**Affiliations:** 1 Institute of Radiopharmaceutical Cancer Research, Helmholtz-Zentrum Dresden-Rossendorf, Bautzner Landstraße 400, 01328 Dresden, Germanyc.haase@hzdr.de (C.H.-K.); r.wodtke@hzdr.de (R.W.); k.kopka@hzdr.de (K.K.); 2 Faculty of Chemistry and Food Chemistry, School of Science, TUD Dresden University of Technology, Mommsenstraße 4, 01062 Dresden, Germany; 3 Institute of Bioanalytical Chemistry, Centre for Biotechnology and Biomedicine (BBZ), Faculty of Chemistry and Mineralogy, Leipzig University, Deutscher Platz 5, 04103 Leipzig, Germany; 4 Institute of Radiopharmaceutical Cancer Research, Research Site Leipzig, Helmholtz-Zentrum Dresden-Rossendorf, Permoserstraße 15, 04318 Leipzig, Germany; 5 Department of Chemistry, Faculty of Chemistry and Chemical Engineering, Babeș-Bolyai University, Str. Arany Janos Nr. 11, RO-400028 Cluj-Napoca, Romania

**Keywords:** cyclooxygenase, dicarbadodecaborane, iodine-123, SPECT imaging

## Abstract

Dicarbadodecaboranes (12) (carboranes) are versatile molecular building blocks with unique properties, which allow the expansion of classical medicinal-chemical space. To enable single-photon emission computed tomography (SPECT) imaging of cyclooxygenase-2 (COX-2), we investigated the feasibility of introducing iodine-123 into *nido*-indoborin **1**, a *nido*-carborane analog of indomethacin with potent and selective cyclooxygenase-2 inhibitory activity. An electrophilic iodination strategy afforded two regioisomers, **2a** and **2b**, bearing the iodine at the carborane cluster. Compared to *nido*-indoborin, a reduced COX-2 inhibition potency and selectivity were observed, with **2b** exhibiting the more favorable inhibition profile. Radiosynthesis of **[^123^I]2b** was achieved by *N*-chlorosuccinimide–mediated electrophilic substitution of **1**, and conditions were optimized, leading to an isolated radiochemical yield of 4%. While the radiotracer displayed high stability in phosphate buffer, ester hydrolysis was observed in human plasma and murine liver microsomes with no significant deiodination in vitro. Cell uptake studies indicated partial COX-2–dependent accumulation but also revealed substantial non-specific uptake and unexpected enhancement of radiotracer uptake in the presence of carborane-based blocking agents. In vivo pilot imaging studies in mice bearing U87 xenografts showed renal and hepatobiliary clearance without measurable tumor accumulation but evidence of deiodination over time. Overall, iodination was feasible, but the resulting compounds lacked the required COX-2-selective tumor accumulation for further radiotracer development.

## 1. Introduction

**Figure 1:**
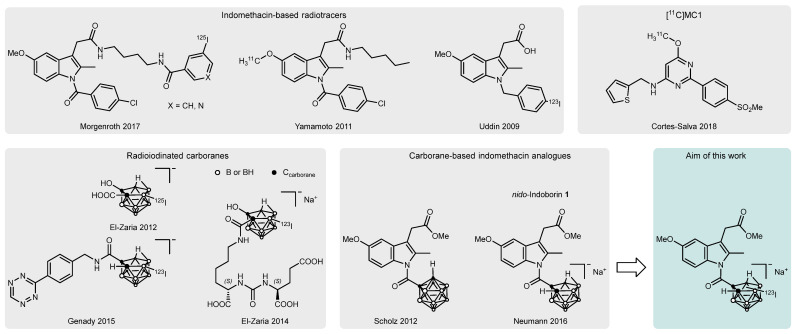
Examples of indomethacin-based COX-2 targeted radiotracers (**top left**) [35,36,43], radioiodinated carboranes (**bottom left**) [7,8,54], and carborane analogs of indomethacin (**bottom middle**) [50,51,55], leading up to the aim of this work (**bottom right**). The structure of [^11^C]MC1 [56] is shown as an example of a promising ^11^C-labeled radiotracer for COX-2 (**top right**).

Three-dimensional boron-carbon cages have become increasingly attractive pharmacophores in medicinal chemistry, enabling fine-tuning of solubility, steric properties, stability, and charge. In particular, these scaffolds can engage in atypical molecular interactions such as dihydrogen bonds, arising from the boron hydride framework. As the most prominent, the icosahedral *closo*-dicarbadodecaborane(12) or *closo*-carborane cluster is composed of 10 boron and 2 carbon vertices (formula C_2_B_10_H_12_). The positions of the C−H units define the increasingly hydrophobic *ortho* (1,2), *meta* (1,7), and *para* (1,12) carborane isomers. Especially, the *ortho* clusters are susceptible to nucleophilic abstraction of one boron vertex, using Lewis bases such as TBAF, CsF or KOMe, generating the more hydrophilic anionic *nido*-carboranes (dodecahydrido-*nido*-undecaborates(1−), [C_2_B_9_H_12_]^−^) [1,2,3,4]. Their availability as a precursor for radiolabeling reactions, especially using radioiodine, has been explored in the past but remains underutilized (Figure 1, bottom left) [5,6,7,8]. With respect to the attachment of radiohalogens to tumor-targeting peptides and proteins, polyhedral borane anions, including *nido*-carboranes, were discussed and explored as potential linker units. This interest was motivated by the higher strength of boron–halogen bonds compared to carbon–halogen bonds and the absence of respective enzymatic systems for cleavage of such motifs due to their xenobiotic nature [6,8,9]. This prompted us to explore the utility of carboranes for the development of molecular imaging probes toward cyclooxygenase-2, a long-standing focus of research in our group [10,11,12,13,14].

Cyclooxygenases (COXs) are heme-dependent oxidoreductases that catalyze the oxidation and concerted cyclization of arachidonic acid to prostaglandin G_2_ (PGG_2_), placing them at a key regulatory point in prostanoid signaling. COX-1 is constutitively expressed in nearly all tissues and mainly functions as a housekeeping enzyme, regulating processes such as thrombocyte homeostasis and kidney physiology. Its isozyme COX-2 is inducible and part of proinflammatory signaling, pain potentiation, and fever stimulation [15,16]. Importantly, constant COX-2 induction can promote the development of malignancies from chronic inflammation, and its overexpression has been associated with a range of cancer entities [17,18,19]. By promoting tumor growth, metastasis, tissue invasion and resistance to therapy, COX-2 contributes to poorer clinical outcomes [20,21,22]. A COX-2 radiotracer would therefore help non-invasive characterization of expression, improving early diagnosis of potentially malignant tissue, and giving a basis for therapy decisions [23,24]. Most radiotracers targeting COX-2 thus far have not met the requirements for clinical translation, and the field remains a topic of research. Key challenges to overcome include metabolic instability, non-specific accumulation, and insufficient tumor uptake [24,25]. Several radioiodinated probes have been developed to address the limitations of current COX-2 radiotracers associated with insufficient half-life of the radionuclide. Most COX inhibitors show time-dependent enzyme inhibition profiles [26]. Further, their high lipophilicity, which is required to reach the intracellular target, facilitates non-specific accumulation. The long half-lives of iodine isotopes in use for nuclear imaging (^123^I: 13.2 h, ^124^I: 4.2 d) promise detection at later time points after an adequate period of equilibration and clearance, respectively, with the aim of reducing the background signal [27]. The first radioiodinated COX-2 inhibitors were developed in 2005 using celecoxib as a lead structure [28,29] and were subsequently tested in both inflammation and tumor models [30,31]. Additionally, a strategically placed iodine substituent may improve the isoform selectivity by harnessing the steric bulk of the large halogen as reported for radioiodinated nimesulide analogs by Yamamoto and coworkers [32,33]. Further radioiodinated COX-2 inhibitors based on pyricoxib as presented by Tietz et al. [34] and indomethacin as presented by Uddin et al. [35] and Morgenroth et al. [36] highlight the opportunities of radioiodinated probes for COX-2 imaging. The 4-methoxypyrimidine-based radiotracer [^11^C]MC1 (Figure 1, top right) is, however, currently the most promising and is enrolled in clinical trials for COX-2 imaging in neuroinflammatory diseases, holding also potential for tumor detection. In xenograft models, [^11^C]MC1 demonstrated uptake in HT-29 tumors, but not in PANC-1 and MDA-MB-231 tumors [37,38,39].

As a non-selective COX inhibitor, indomethacin belongs to the group of non-steroidal anti-inflammatory drugs (NSAIDs) and has been used as a scaffold for the development of COX-2 fluorescent imaging agents and radiotracers (Figure 1, top left). It is modifiable at the acetic acid sidechain by ester or amide formation, yielding COX-2 selective compounds and giving a possible point of attachment for radio- or fluorescent labels [35,36,40,41,42,43,44]. Reports are in accordance with the fact that these substituents protrude from the COX substrate channel, which explains the tolerance of their steric bulk. To impart COX-2 selectivity to NSAIDs, carboranes have been applied as bioisosteric replacements for phenyl moieties, taking advantage of the slightly larger hydrophobic side pocket within the COX-2 substrate channel [45,46,47,48,49]. We reported the synthesis of *closo*- and *nido*-carborane analogs of indomethacin and found *nido*-indoborin **1** (Figure 1, bottom middle) as a substantially more potent methyl ester derivative bearing an *ortho*-*nido*-carborane in place of the 4-chlorophenyl moiety [50,51,52]. Of note, the conversion of the *ortho*-*closo* derivative into the *nido*-indoborin analog, which showed higher COX inhibitory potency, occurred even under buffered in vitro assay conditions. This observation led to the initial development of *nido*-indoborin and highlights the facile deboronation of *ortho*-*closo* clusters to the corresponding *nido* species. The phenyl congener indomethacin exhibits time-dependent, but reversible COX inhibition [26]. **1** is therefore regarded as a reversible inhibitor of COXs, as it has no reactive group commonly called a warhead. Interestingly, the relocation of the negative charge from a carboxylic acid to the *nido*-carborane led to a changed binding mode of **1** within the active site of COX-2 [50,51,52]. As the orientation of *nido*-indoborin within the substrate channel is inverse compared to indomethacin, its esters and amides, we hypothesize that modifications at the carborane moiety should be possible without impairing the inhibitory profile and that the addition of a large substituent or radiolabel might even improve COX-2 selectivity. Combined with the advantages of radioiodine in the context of COX-2 radiotracers and the metabolic inertness of the carborane cage, the *nido*-indoborin scaffold represents a promising precursor.

This work aimed at investigating an iodinated derivative of *nido*-indoborin **1** for its COX-2 inhibitory properties and, as an iodine-123–labeled counterpart, for its suitability as a radiotracer for functional imaging of COX-2 using single-photon emission computed tomography (SPECT). We previously reported preliminary data in a conference abstract [53]. The current study extends this work by providing further insight into stability and the proposed metabolism of the radiotracer, as well as further cellular and initial in vivo results.

## 2. Results and Discussion

### 2.1. Chemical Synthesis

**Scheme 1:**
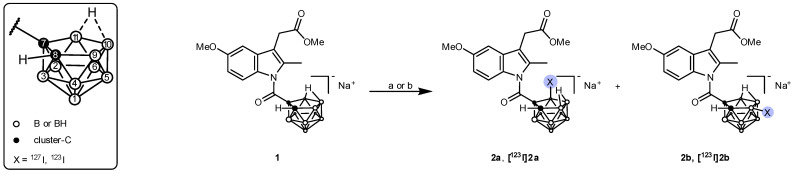
Reaction conditions of (radio)iodination and *nido*-carborane numbering scheme. **Left** (box): Numbering scheme of 7-substituted *ortho*-*nido*-carboranes. **Right**: (Radio)iodination of **1**. (a) X = ^127^I (i) 20 eq. AcOOH, 1 eq. NaI, CH_3_OH, rt, 2–4 h. (ii) 6 eq. Na_2_S_2_O_5_. (b) X = ^123^I (i) 1 eq. NCS, [^123^I]NaI, H_2_O, rt, 10 min. (ii) 20 eq Na_2_S_2_O_5_. Only one enantiomer and tautomer of all compounds are shown.

**Figure 2:**
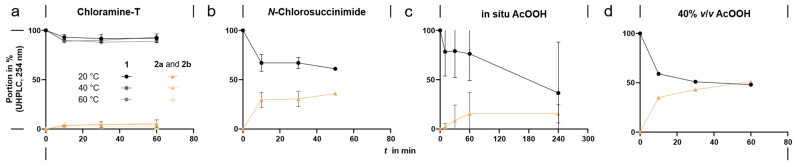
Optimization of the reference synthesis. Iodination conditions tested in nanomole scale using different oxidants: (**a**) CAT, (**b**) NCS, (**c**) in situ AcOOH, (**d**) 40% *v*/*v* AcOOH. Product formation (**2a** and **2b** combined, orange triangles) and consumption of starting material **1** (black circles) over time are depicted as the mean ± SD of peak areas as determined by absorption at 254 nm in ultra-high performance liquid chromatography (UHPLC) analysis. Lighter shades of symbols in (**a**) represent increasing reaction temperatures. *n* = 4 (CAT 20 °C, in situ AcOOH), *n* = 2 (NCS), *n* = 1 (all others).

As described earlier by us, *nido*-Indoborin **1** was synthesized with some minor modifications [51,55]. We envisaged the synthesis of the iodo-*nido*-carborane derivative starting from racemic **1** in an electrophilic substitution reaction. Under mild conditions, *nido*-carboranes are readily halogenated [57,58,59]. The drawback of the reaction is the possible creation of regioisomers—similar to iodination by electrophilic aromatic substitution of classical phenyl-based compounds. The presence of two potential iodination positions, *B*-9 and *B*-11, on the open face of the *nido*-carborane adjacent to the carbon vertices is responsible for this behavior (Scheme 1). Unsymmetrical substitution of the carborane cluster will lead to the selective formation of one isomer. To optimize the reaction, different oxidants and conditions for the transformation of iodide to electrophilic species and subsequent halogenation were tested at the nanomole scale. The reaction progress was monitored by UHPLC-MS analysis of an aliquot at given time points. Chloramine-T (CAT) did not convert substantial amounts of **1** into its iodinated form at temperatures between 20 and 60 °C (Figure 2a). Next, we tested *N*-chlorosuccinimide (NCS) as well as in situ generated peracetic acid (AcOOH), which was formed by mixing acetic acid and hydrogen peroxide 2 h prior to the reaction start to account for equilibration (Figure 2b,c). Using either NCS or in situ generated AcOOH led to a mixture of regioisomers **2a** and **2b** within 1 h (numbering refers to elution order on HPLC); however, the use of in situ generated peracetic acid was prone to inconsistent conversion results due to high variance in the formation of AcOOH and degradation of **1** as a result of the presence of residual H_2_O_2_. Therefore, the application of commercially available AcOOH 40% *v*/*v* was tested and gave not only fewer degradation products but also the highest conversion to **2a** and **2b** (Figure 2d). The use of AcOOH 40% *v*/*v* was hence preferred for further experiments. Finally, the reaction conditions were transferred to a micromole scale, which required further adjustment, i.e., 20 molar equivalents (eq.) of AcOOH had to be used for efficient oxidation of iodide, while only one equivalent of sodium iodide was used to avoid double iodination (Scheme 1). Quantitative conversion of **1** to **2a** and **2b** in a ratio of approx. 1:5 (HPLC, 254 nm) was achieved within 2–4 h (Supplementary Information Figure S1).

While under the optimized synthesis conditions, **1** could be quantitatively converted into mixtures of regioisomers **2a** and **2b**, attempts to separate and isolate the pure compounds proved challenging. On C18-endcapped HPLC columns, a mixture of **2a** and **2b** (Supplementary Information Figure S2) in ratios between 1:6 and 1:20 was obtained. Further, removal of HPLC eluents containing trifluoroacetic acid (TFA) via freeze-drying led to the formation of degradation products compromising the purity to < 80%. Among the impurities, indolylacetic acid derivatives **3a** and **3b** could be identified (Supplementary Information Figures S12–S15). During distillation, TFA and water form an upper azeotrope which contains approx. 21% *w*/*w* of TFA [60,61]. Although this behavior cannot directly be applied to freeze drying, a concentration of the acid during this process might likely have caused the ester hydrolysis in this case. Therefore, HPLC fractions were subjected to solid phase extraction (SPE) using a C18-endcapped cartridge and subsequent elution with ethanol, which was removed under reduced pressure. The compounds were still obtained at a purity of 60–85% and were prone to degradation when stored at room temperature without an inert atmosphere. The exact nature of the impurities could not be elucidated. Interestingly, the unsubstituted compound **1** exhibited remarkable stability if stored under similar conditions. The introduction of the anionic *nido*-cluster had a stabilizing effect on the amide bond by counterbalancing the electron-withdrawing effect of the carbonyl group toward the indole-nitrogen atom [51]. So, it may be reasoned that this effect is somewhat counteracted by iodine substitution of the carborane, as it lowers the electron density of the cluster, in turn weakening the amide bond. A pentafluorophenyl-endcapped column was employed, which allowed complete separation of the isomers for identification in radio-HPLC experiments directly using the collected HPLC fractions. Further attempts to stabilize the isolated products are currently being undertaken. All characterization so far had to be conducted using products **2a** and **2b** containing varying amounts of impurities, which are declared accordingly (for further information, see Supplementary Information Table S1).

**2b** was characterized using ^1^H, HSQC, ^11^B NMR and HRMS, which collectively proved that the carborane cage was carrying the iodine substituent. In ^1^H NMR, the splitting and multiplicity of aromatic and aliphatic protons are retained between **1** and **2b**, supporting iodination at the carborane cluster. Most notable changes occurred for the carborane cage C–*H* signal: compared to precursor **1**, the signal experienced a downfield shift in **2b** (from 1.59 to 2.86 ppm, Supplementary Information Figure S5). ^11^B and HSQC-derived ^13^C signals could not contribute further information to assign the exact iodination position (Supplementary Information Figures S6–S8). The assignment of the B–I signal in theory would be possible by comparison of the proton-coupled and -decoupled ^11^B NMR spectra, but requires pure samples and higher substance amounts [8,54,62]. HRMS analysis in negative mode showed the expected signal and isotopic pattern for the intact iodinated compound. Further, HRMS analysis showed the fragmentation product 7-carboxy-9-iodo-nonahydrido-10,11-µ-hydrido-7,8-dicarba-*nido*-undecaborate(1–) or its 11-iodo-isomer with the expected isotopic pattern (*m*/*z* calculated 304.0683, found 304.0681), confirming the iodination at the *nido*-carborane cluster. Elucidation of the exact position of iodine in the cluster was not successful based on NMR or HRMS, however. The *C*-7 substitution by the carboranyl group creates an electronic and steric situation within the C_2_B_3_ plane of the *nido*-carborane that favors iodination of *B*-9 over *B*-11 (Scheme 1, right) [8,63,64]. Low amounts of **2a** allowed characterization by HRMS only, which confirmed the iodination at the cluster, but NMR could not be successfully performed due to low isolated substance amounts.

### 2.2. COX Inhibitory Activity

Compounds **2a** and **2b** were tested in an enzymatic fluorescence-based COX inhibition assay using the selective COX-1 inhibitor SC-560 and the COX-2 selective inhibitor celecoxib as reference. At 100 µM, isomer **2b** exhibited higher inhibition than **2a** towards both isozymes (Table 1). COX-2 inhibition potency was more pronounced for **2b** (HPLC-purity 91%, *IC*_50_ 4.42 µM) compared to **2a** (HPLC-purity 75%, *IC*_50_ 42.1 µM), and in both cases, lower compared to the native *nido*-indoborin **1** (*IC*_50_ of 0.91 µM). *IC*_50_ towards COX-1 was found to be slightly lower for **2a** (10.6 µM) than for **2b** (15.8 µM). In turn, the COX-2 selectivity indices (SI) of **2a** (0.25) and **2b** (3.6) were markedly decreased compared to *nido*-indoborin **1** (SI > 109). Taken together with the impurities contained within the samples, the reported values have to be treated with utmost care and are mere indications of the actual inhibitory potency of compounds **2a** and **2b**. For the same reason, the inhibition kinetics could not be assessed. Based on the known properties of indomethacin and *nido*-indoborin, **2a** and **2b** are nevertheless assumed to act as reversible COX inhibitors. Despite these experimental limitations, the observed inhibition profile remains surprising: an increased size of the molecule generally promotes COX-2 over COX-1 inhibition, using the larger hydrophobic side pocket to the substrate channel present in COX-2, but not COX-1 [16,65]. Further, Neumann et al. found that **1** can access a sub-pocket in COX-2 formed upon rotation of Leu531 [51]. This cavity also accommodates the benzothiazine of oxicams and is known to be important for celecoxib binding [66,67]. Blobaum et al. proposed that substitution of the 2′-methyl group of indomethacin by trifluoromethyl drives the methoxyindole moiety deeper into the Val523 side pocket, thereby improving COX-2 selectivity through additional engagement of the Leu531 pocket [68]. On this basis, we hypothesized that the iodine substituent at the carborane cage might similarly occupy the latter pocket and promote a deeper insertion of the methoxyindole into the Val523 pocket, ultimately enhancing inhibitory potency towards COX-2. Contrary to our expectations, the COX assay results did not confirm this hypothesis. However, the only slightly lower COX-2 inhibitory potency of **2b** compared to **1** encouraged us to proceed with the radioiodination in this project.

### 2.3. Radiosynthesis

Due to the higher COX-2 inhibition potency of **2b** compared to **2a**, the radioiodination reactions were aimed to produce and isolate **[^123^I]2b**. Labeling with iodine-123 was performed via electrophilic substitution, similar to non-radioactive iodination starting from 0.1 nmol precursor **1**. Reaction conditions were optimized successively in independent experiments regarding oxidant, solvent, temperature, reaction time, and pH (Table 2, entries A–S) employing activities of approximately 1–83 MBq [^123^I]NaI in 0.02 M NaOH per reaction. Contrary to non-radioactive iodination, a range of byproducts was observed for all tested conditions. Total radiochemical conversion (RCC_total_) is therefore distinguished from RCC to product **[^123^I]2b**. The oxidant NCS was superior to CAT and AcOOH (Table 2, entries A–C), while water was found to be the most suitable solvent compared to CH_3_CN and dimethyl sulfoxide (DMSO) (Table 2, entries D–F). The reaction using NCS in water was conducted at temperatures between 20 °C and 90 °C, showing an optimum RCC to **[^123^I]2b** at 40 °C (Table 2, entries G–K). Since conversion to **[^123^I]2b** only increased marginally and mainly the formation of side products was enhanced, further optimizations were conducted at room temperature (20 °C). Increasing reaction time from 10 min up to 120 min gave comparable RCC to **[^123^I]2b** (Table 2, entries L–O), so that a 10 min reaction time was used throughout. We further investigated adjusting the pH of the [^123^I]iodide solution by the addition of H_3_PO_4_ because, on the one hand, aqueous NaOH solution added as solvent of [^123^I]NaI was expected to be one reason for side product formation due to the hydrolysis of the labile amide bond. On the other hand, the reactivity of NCS is markedly increased in acidic media, promoting the formation of iodine monochloride, which rapidly forms hypoiodous acid [70,71], acting as the actual electrophilic iodine species. Hence, the reaction was conducted at different concentrations of dilute phosphoric acid solutions between 3 mM and 42 mM (Table 2, entries Q–S). Using 10 mM H_3_PO_4_ (entry R), which approximately adjusts the pH of the reaction mixture to pH 5 after addition of radioiodine, gave the most suitable RCC to **[^123^I]2b** with a notable improvement compared to water (entry P). Of note, further experiments were performed to investigate the concentration dependence for NCS and **1** without the addition of H_3_PO_4_. When equimolar concentrations of NCS and precursor **1** were lowered by a simultaneous 1:1 dilution, both total RCC and RCC to **[^123^I]2b** declined markedly below concentrations of 1 mM. When the concentration of NCS was kept constant at 2 mM, and only **1** was diluted instead, nearly constant RCC to **[^123^I]2b** and total RCC were observed for concentrations as low as 0.125 mM of **1** (Supplementary Information Figure S17a).

Scaling up the reaction with respect to starting activity showed that higher amounts of [^123^I]iodide and hence a decreased excess of **1** resulted in a decline of RCC (Supplementary Information Figure S17b). In line with this, the addition of non-radioactive iodide as a carrier [36], failed to improve RCC. The initial concentration of the precursor **1** was therefore kept at 2 mM.

Finally optimized conditions for radiosynthesis of **[^123^I]2b** comprised the use of 10 mM aqueous H_3_PO_4_, 2 mM NCS, 2 mM precursor **1**, and 10 min reaction time. The conditions were applied to reactions starting from up to 530 MBq [^123^I]iodide in a total volume of 60 µL. Purification was performed by semipreparative HPLC and SPE using C18-endcapped silica-based column materials. Final elution of the SPE cartridge with ethanol and evaporation under reduced pressure and a stream of nitrogen gas provided **[^123^I]2b** in 1–4% radiochemical yield (RCY). In certain cases, multiple reaction batches were performed in parallel and united before HPLC purification to increase the activity yield. In this manner, upscaled reactions starting from 60 to 530 MBq per batch and a total activity of up to 4300 MBq [^123^I]iodide provided **[^123^I]2b** in an activity of up to 62 MBq, radiochemical purity (RCP) > 94%, and *A*_m_ > 21 GBq/µmol, within a synthesis time of approx. 90 min (*n* = 13). The molar activity of iodine-123 produced via the ^124^Xe(p,pn) reaction is very near the maximum theoretical molar activity of 8771 GBq/µmol (reported values of 8695 GBq/µmol) [72]. However, in this case, the low amounts of obtained radiotracer precluded a more exact determination. The identity of the iodine-123–labeled species was confirmed by co-injection with the non-radioactive reference compound **2b** (Supplementary Information Figure S18).

### 2.4. Stability Studies

**Figure 3:**
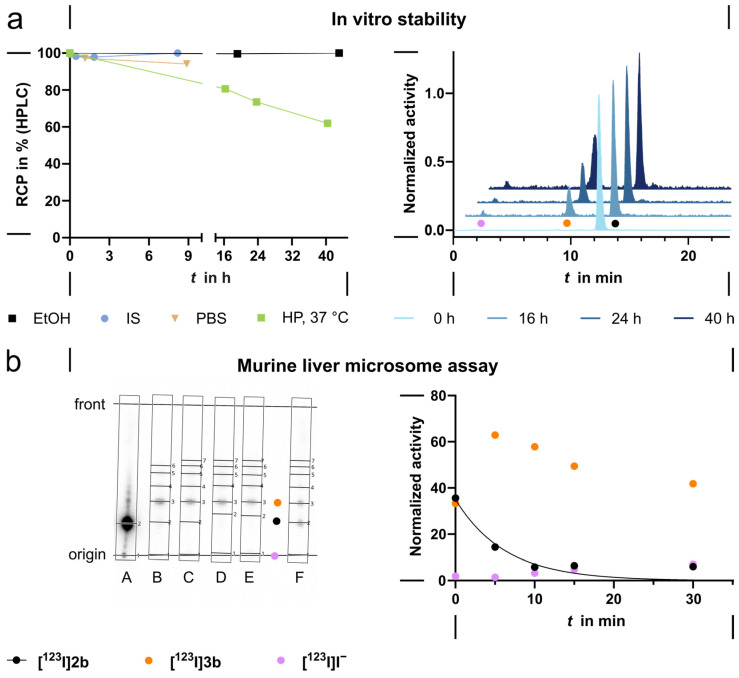
Stability studies of **[^123^I]2b**. In vitro stability studies (**a**) and murine liver microsome assay (**b**). (**a**) Left: Incubation of **[^123^I]2b** in ethanol (black square), 0.9% saline solution (IS, blue circle), PBS (orange triangle), and HP (green square) over 8 h (0.9% saline solution, PBS) and 40 h (EtOH, HP), respectively. Incubations were conducted at rt, unless stated otherwise. (**a**) Right: Radio-HPLC analysis after plasma incubation of **[^123^I]2b** (black circle, t_R_ = 12.6 min) showing increasing formation of **[^123^I]3b** (orange circle, t_R_ = 8.8 min) and [^123^I]iodide (purple circle, t_R_ = 1.5 min) over 40 h. (**b**) Left: Radio-TLC of murine liver microsome samples: reference **[^123^I]2b** (A), samples withdrawn from oxidative conditions after 5 min (B), 10 min (C), 15 min (D), and 30 min (E), control without added NADPH withdrawn after 60 min (F). (**b**) Right: Time course of **[^123^I]2b** (black), **[^123^I]3b** (orange), and [^123^I]iodide (purple) in murine liver microsomes. Half-life of **[^123^I]2b** was calculated to be 4.3 min (one-phase decay, y = y_0_ × e^−kx^).

Stability studies of **[^123^I]2b** were conducted in ethanol (EtOH), 0.9% saline solution, phosphate-buffered saline pH 7.4 (PBS), and human plasma (each containing 5% *v*/*v* EtOH). The tracer was stable in EtOH over 40 h and over 8 h in 0.9% saline solution and PBS (Figure 3a). When incubated in human plasma (HP) at 37 °C, RCP decreased to 62% over a course of 40 h. The degradation product could be identified via co-injection on HPLC as the respective 2-(3-indolyl)-acetic acid derivative **[^123^I]3b** resulting from hydrolysis of the methyl ester group (Supplementary Information Figure S19). Several plasma enzymes can principally lead to this transformation, like plasma esterases (acetylcholinesterase, butyrylcholinesterase, paraoxonase 1) [73,74], but the responsible enzyme was not elucidated within this study. Only a minor fraction of [^123^I]iodide (2% after 40 h) was detected, proving the stability of the B–I bond in vitro.

In a murine liver microsome assay (Figure 3b), **[^123^I]2b** was converted to five more hydrophilic radiometabolites detected by radio thin layer chromatography (radio-TLC) over a course of 30 min incubation at 37 °C. The oxidative metabolism by cytochrome P450 (CYP) enzymes is dependent on the cofactor nicotinamide adenine dinucleotide phosphate (NADPH), and a control reaction was conducted in the absence of NADPH. Most notably, the whole spectrum of metabolites formed did not differ between the control and oxidative conditions. The major metabolite (63% after 5 min) could be identified as the indolyl acetic acid derivative **[^123^I]3b** via radio-TLC (Supplementary Information Figure S21). Since this metabolite is also found as the main derivative in the absence of NADPH (33% after 60 min), the presence of murine carboxylesterases (mCES) in the liver microsomes likely contributes to this initial metabolic step of methyl ester hydrolysis [75]. Consistent with the results obtained in human plasma, **[^123^I]2b** did not show substantial deiodination (1% increased to 7%). Without the addition of NADPH (control), free [^123^I]iodide amounted to only 2% over a course of 60 min, indicating that deiodination is mainly a result of CYP activity.

As carboxylesterase (CES) is essentially absent from human plasma, the results from the liver microsome assay indicate that **[^123^I]2b** can be a substrate for various esterases. Of note, human CES1 (hCES1) is mainly expressed in liver and lung, and hCES2 is limited to intestines and kidneys, while murine liver cells express orthologs of both enzymes. The substrate specificity of hCES1 for esters featuring a short-chained alcohol and a bulky acid makes the involvement of this isoform here more likely [76,77]. This conclusion is further supported by the work of Takahashi et al., who synthesized an array of indomethacin ester prodrugs and meticulously characterized hCES1/2 selectivity. For short-chained, unbranched alcohols, hydrolytic rates were found to be much higher in human liver microsomes (i.e., hCES1) compared to human intestinal microsomes (i.e., hCES2) [78,79]. The discrepancy in the amount of the main metabolite **[^123^I]3b** between oxidative and non-oxidative conditions indicates a contribution to methyl ester hydrolysis by CYPs as described by Peng et al. [80]. Under oxidative conditions, a maximum of **[^123^I]3b** was detected after 5 min, while other metabolites increased over time. This indicates further metabolization, most likely through CYP-mediated hydroxylation followed by *O*-demethylation of the methoxy group and/or *N*-deacylation of the indole, which is also attributed to the activity of carboxylesterase. These are the main phase I metabolic pathways described for the phenyl analog indomethacin [81,82,83]. Taking the EtOH concentration in the assay into account (0.2% *v*/*v* or approx. 43 mM), mCES may also catalyze transesterification reactions [84,85]. Taken together, this would explain four of the radioactive metabolites found in the absence of NADPH (Supplementary Information Figure S20). No structural elucidation was undertaken, however. On the other hand, this assumption would not allow for metabolites formed only under the influence of CYPs. Contrary to human blood, mouse blood contains mCES1C, which would suggest a more rapid metabolism of **[^123^I]3b** in the blood of mice, both in vitro and in vivo [76,77,86]. In a previous study investigating the *closo*-carborane-based leads of *nido*-indoborin **1**, it was shown that cleavage of the methyl ester to the carboxylic acid derivatives resulted in less potent inhibitors of COXs [55]. However, Neumann et al. predicted similar binding properties for the *nido*-carborane **1** and its free carboxylic acid by molecular docking [51]. Thus, the COX-2 inhibition potency of **2b** and **3b** might be comparable, as well.

The distribution coefficient logD_7.4_ of **[^123^I]2b** was determined to be 1.35 using the shaking flask method. This value should allow membrane passage, though the negative charge of the molecule might impair that process. Comparison with the logD_7.4_ of indomethacin of 0.91 (determined using the shaking flask method) shows that the negative charge of the *nido*-carborane, the methyl ester formation and iodination do not significantly change the distribution properties at pH 7.4 [87]. As logD_4.5_ of indomethacin rises to 3.86, the acidic NSAID can cross cell membranes with ease, as is intended for that group of anti-inflammatory drugs taking advantage of the acidic extracellular medium in inflamed tissue [87]. The *nido*-carborane unit is a weaker base than the acetic acid derivative (p*K*_a_ = 2.98 (exp.), −4.6 (calc.) [88]) and protonation to form a neutral species is disfavored under physiological conditions. For comparison, logD_7.4_ of the precursor **1** was previously determined using an HPLC-based method to be approximately 2.0 [69].

**Figure 4:**
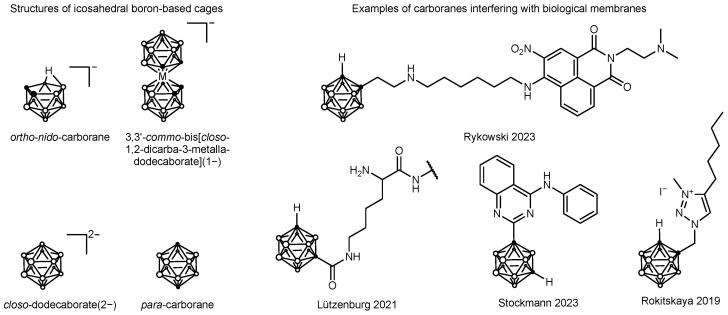
Carborane structures reported in BSA binding studies and as membrane-interacting compounds. (**Left**): Classes of icosahedral boron-based cages employed in BSA binding studies [89]. M is exemplary for a trivalent metal cation. (**Right**): *closo*-Carboranes that were reported to interfere with biological membranes using varying mechanisms [94,95,96,97].

Plasma protein binding was measured to be 96% using human plasma and Amicon spin filters (molecular weight cut-off 10 kDa), which is close to the value of 90% reported for indomethacin [83]. Strong and specific interactions between monoanionic bis(dicarbollide) sandwich complexes and bovine serum albumin (BSA) have been reported (see Figure 4 left). Neutral *para*-*closo*-clusters were interacting to a lesser degree, but Goszczyński et al. did not investigate *nido*-carboranes [89]. Dodecaborate(2−) dianions, however, showed far lower affinity to albumin. It can hence be reasoned that *nido*-cluster **[^123^I]2b** unites features of monoanionic metallacarboranes and *closo*-carborane and that plasma protein binding is based on an interaction with albumin. Further, indomethacin is reported to bind to fatty acid binding sites FA1 and FA7 [90,91,92,93].

### 2.5. In Vitro Cell Studies

To determine the role of COX-2 for cell uptake of **[^123^I]2b**, in vitro studies with U87 human glioblastoma cells overexpressing COX-2 were conducted (U87). For the determination of COX-2–independent uptake, reference cell lines U87^COX−2KO^ and human glioblastoma cell line U251 were used. U87^COX−2KO^ was derived from U87 by CRISPR/Cas9-mediated knockout of COX-2, while U251 exhibits no to low expression of COX-2 [98,99,100,101]. For blocking, COX-2 inhibitor celecoxib, non-selective COX inhibitor indomethacin (COX-1 IC_50_ = 0.38 µM, COX-2 IC_50_ = 18.2 µM; determined under the same assay conditions as **2b**) [102], precursor **1** and non-radioactive reference **2b** were used at 100 µM.

**Figure 5:**
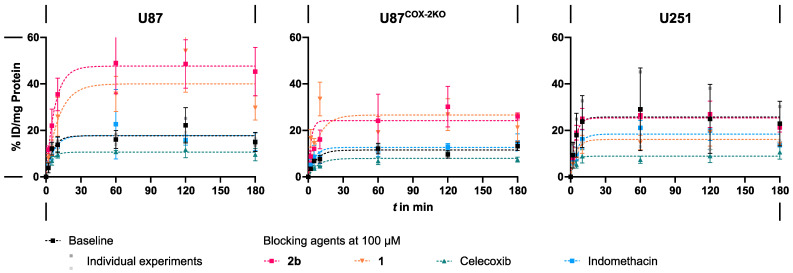
Cell studies of **[^123^I]2b**. In vitro cell uptake of **[^123^I]2b** (0.10–0.12 MBq/mL, > 18.6 GBq/µmol, RCP ≥ 95%) in U87, U87^COX−2KO^, and U251 cells over 180 min. Cells were preincubated with 100 µM of the corresponding blocking substance (baseline—black square, **2b**—red square (purity 86% (HPLC, 254 nm)), **1**—orange triangle, celecoxib—green triangle, indomethacin—blue square) for 30 min before addition of the radiotracer. Results are given as mean ± SD, represented as % initial dose (ID) per mg protein (*n* = 3; *n* = 6 for baseline). Means of individual experiments are shown for baseline uptake as gray squares (SD omitted). Curves were fitted to a one-phase association model (dashed lines, *y* = *y*_0_ + (*a* − *y*_0_) × (1 − e^−*kx*^)).

In the absence of the blocking agents (baseline conditions), saturation of cellular uptake was reached within 30–60 min, amounting to 15–20% of the initial dose (ID) per mg protein in U87, and 10–15% in U87^COX−2KO^ (Figure 5). Cell uptake in U251 did not reproducibly reach a plateau between 15% and 40% ID/mg protein. Celecoxib blocked the uptake of **[^123^I]2b** in U87 to some extent, but remaining unspecific binding was observed. In U87^COX−2KO^ and U251 cells, celecoxib diminished cell uptake to a lesser degree. For all cell lines, activity uptake plateaued at 5–10% ID/mg protein, which supports the COX-2-dependent cell binding of **[^123^I]2b**, but shows unspecific interactions as well. Indomethacin showed no blocking effect in any of the three cell lines, with uptake levels being comparable to baseline. This suggests COX-1–independent uptake of **[^123^I]2b**. Interestingly, blocking agents **1** and **2b** (HPLC-purity of 86%) led to an increased cell uptake in both U87 and U87^COX−2KO^ cells. Native *nido*-indoborin **1** elevated uptake to 30–45% ID/mg protein in U87 and 20–30% ID/mg protein in U87^COX−2KO^. Iodinated compound **2b** led to increased cell uptake of 45% and 25% ID/mg protein in U87 and U87^COX−2KO^, respectively. Relative to the baseline, the uptake was nearly doubled in both U87 and U87^COX−2KO^ cells by the *nido*-carborane–bearing compounds. No similar observation was made for U251.

To explain the influence of **1** and **2b** on the cell uptake of **[^123^I]2b**, two hypotheses were tested: first, that compounds **1** and **2b** inhibit members of the multidrug resistance (MDR) protein family; and second, that membrane permeabilization or facilitated membrane passage is achieved by compounds **1** and **2b**. Initially, both U87 and U251 do not express high quantities of MDR protein without stimuli. The most common MDR-associated ATP-binding cassette (ABC) transporters, permeability glycoprotein (P-gp, ABCB1), MDR protein 1 (MRP1, ABCC1), and breast cancer resistance protein (BCRP, ABCG2), are only found at low levels of mRNA and protein expression. Additionally, BCRP appears to be mostly localized at the nuclear membrane of glioblastoma cell lines [103,104,105,106,107]. Being a substrate to an MDR ABC transporter would greatly hinder the applicability of a COX-2 radiotracer because of the intracellular location of the target enzyme. In our experiments, neither the third-generation noncompetitive P-gp inhibitor and BCRP substrate tariquidar (2 µM) nor the competitive P-gp inhibitor and MRP1 inhibitor and modulator verapamil (100 µM) affected the cellular uptake of **[^123^I]2b** in a similar manner to **1** and **2b** (Supplementary Information Figure S22) [108,109,110,111]. Hence, involvement of MDR was ruled out as a reason. Further, permeabilization or facilitated membrane passage was considered because **1** and **2b** share anionic head and lipophilic tail components similar to anionic surfactants and may thus cause a similar effect on the cellular uptake of the radiotracer. Given the concentrations of 100 µM of **1** and **2b**, it is considered rather unlikely that a critical micellar concentration (CMC) was reached, which would lead to pore formation and facilitation of transport across the membrane and finally cytotoxicity by the disruption of membrane integrity. In line with this, propidium iodide uptake was not changed by concentrations up to 100 µM of **1** (Supplementary Information Figure S23), so that this process was ruled out. Surfactants are known to facilitate cross-membrane transport at concentrations below CMC by interaction with the phospholipid bilayer [112]. The potential of **1** for an increased calcein efflux from the cell as a sign for membrane leakage was assessed, but also in this experiment, no influence could be observed (Supplementary Information Figure S24). Both hypotheses were thus disproven. Thirdly, considering the high plasma protein binding of **[^123^I]2b**, it is conceivable that the carborane conjugates **1** and **2b** displaced the radiotracer from its binding to BSA, which was present in the cell medium. However, a similar behavior might have been expected for the phenyl analog indomethacin, which also exhibits high plasma protein binding. Further, all hypotheses cannot explain why U251 did not exhibit the same increase in **[^123^I]2b** uptake in the presence of the carborane-based blocking agents. It is therefore concluded that a targeted or cell type-specific mechanism lies at the bottom of this phenomenon, which could, however, not be elucidated within the scope of this study. Other carborane-bearing compounds have been employed for purposes of membrane permeabilization or facilitated transport, such as a triazole-based proton shuttle, 1,8-naphthalimide-based lysosome-membrane permeabilizing compounds, carborane-loaded peptides with improved cargo delivery, and carborane-based ABCG2 inhibitors (Figure 4, right) [94,95,96,97,113,114].

There are several limitations to in vitro cell uptake studies for COX-2 targeted radiotracers due to the target location as well as the rather lipophilic nature of this class of compounds. In order to reach intracellular targets, radiotracers that do not use specific transport mechanisms must cross the cell membrane by means of osmosis. Therefore, they are bound to exhibit a balanced lipophilicity-hydrophilicity, enabling both membrane permeation and solvation in physiologic media. High lipophilicity promotes the adsorption to plastic surfaces, thereby increasing the background activity signal. Glass can also serve as a sorptive surface, a phenomenon previously described for iodinated *nido*-carboranes [6,59]. To minimize potential interactions between tracer and experimental material, our setup was designed to reduce surface binding. The underlying conditions were already advantageous in this regard: anionic species exhibit the lowest propensity to adsorption compared to neutral and cationic compounds, and adsorption is generally reduced in buffered media compared to water. Sorption was further limited by the use of polypropylene cell culture dishes, which have a low tendency for sorption and the presence of BSA in the cell medium, sequestering most of the unbound tracer as shown by plasma protein binding [115,116,117]. Despite these precautions, when working with low concentrations, as in the case of radiotracers, surface saturation effects may occur, leading to a high relative amount of surface-bound analyte, while the absolute amount remains low. In addition, high lipophilicity contributes to non-specific binding to the cell membrane and other organelles. Thus, a rather high background signal in cell studies is not uncommon for COX-2 radiotracers [36,118]. Further limitations arose from the inherent constraints of monolayer cell culture. The growth in a two-dimensional environment forces cells into an unnatural morphology with reduced cell–cell contacts and exposure of the entire cell surface to the surrounding medium. Moreover, quorum-sensing–like effects may alter the behavior of the cells, potentially affecting protein expression levels [119,120]. Additionally, our experimental setup did not allow an assessment of potential cytotoxic effects of the blocking agents beyond visual inspection.

### 2.6. In Vivo Pilot Studies

**Figure 6:**
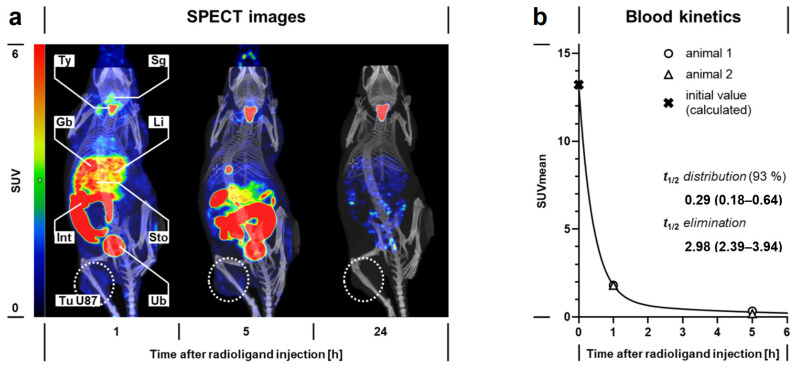
Pharmacokinetic properties of **[^123^I]2b** in mice determined by quantitative SPECT imaging. (**a**) Maximum-intensity projections of U87 tumor-bearing mice at indicated time points after intravenous injection of the radiolabeled compound (n.c.a) administered at an initial dose of 10 MBq (>50 MBq/mL, > 21.7 GBq/µmol, RCP ≥ 97%). (**b**) Estimated pharmacokinetic profile in blood; uptake values of the heart’s blood content were extracted from regions of interest with a volume of 30 mm^3^; the time course of uptake values was analyzed using the non-linear regression model ‘two-phase exponential decay’ with initial and plateau values constrained to 13.2 and 0, respectively; (*t*_1/2_) means of biological half-lives with 68% confidence interval; (Gb) gall bladder, (Int) intestine, (Li) liver, (Sg) salivary glands—sublingual and submandibular, (Sto) stomach, (Tu) tumor, (Ty) thyroid gland, (Ub) urinary bladder, (SUV) standardized uptake value.

Given that in vitro results may not fully resemble in vivo observations made in the case of radiotracers [121,122,123], experiments were conducted in mouse models to give additional insights into the distribution and pharmacokinetics of **[^123^I]2b** in vivo. To corroborate the observations made during in vitro analysis, nude NMRI^*nu*/*nu*^ mice bearing subcutaneous U87 tumor xenografts were injected with **[^123^I]2b**. In SPECT images (Figure 6a), **[^123^I]2b** showed transitional uptake in the liver. A moderate accumulation of activity in the stomach wall, thyroid gland, and the salivary glands (Table 3) points towards radiodeiodination and consequent uptake of [^123^I]iodide via the Na^+^/I^−^ symporter (NIS), known for its high abundance in these tissues [124,125,126]. Quantitative analysis of SPECT images provided tissue-specific time courses of uptake values. Analysis of the region-averaged standardized uptake values (SUVmean) of the heart’s blood content using a biphasic pharmacokinetic model resulted in estimated blood half-lives of 0.29 h for distribution and 2.98 h for elimination (Figure 6b). These values reflect the high plasma protein binding and rapid metabolism in liver microsomes, which were observed for **[^123^I]2b** in vitro. Within 1 h after injection, 8.5% and 48.5% of the initially administered dose were excreted via the renal and the hepatobiliary pathway, respectively (Table 3). This is in contrast to the excretion pathways reported for indomethacin, which shows 20–40% fecal and 60–80% renal elimination as a mixture of indomethacin, its *O*-desmethyl and *N*-deschlorobenzoyl metabolites, as well as glucuronides thereof [81,83]. The distribution and elimination pathways of **1** rather match those reported for an iodine-125–labeled 7-carboxy-decahydrido-9,10-µ-hydrido-7,8-dicarba-*nido*-undecaborate(1–) by El-Zaria et al., describing slow clearance from the blood pool and nearly even elimination via the bile and urine. [54]. Apparently, the presence of the radioiodinated *nido*-carborane induces a shift in the excretion behavior. Interestingly, urine analysis via radio-TLC following SPECT imaging 1 h after injection of **[^123^I]2b** showed the sole excretion of activity as [^123^I]iodide (Supplementary Information Figure S25), which does not match the metabolite profile expected from murine liver microsome experiments and reports on indomethacin. Activity in the liver, gall bladder and gastrointestinal tract may be ascribed to intact **[^123^I]2b** and its metabolites and excretion of [^123^I]iodide via the bile duct and/or the gastric walls. However, the fecal excretion of activity was not analyzed. The observations of radiodeiodination are in accordance with the results of El-Zaria and coworkers. Their work further entailed a *C*-hydroxy-derivative (Figure 1), which exhibited markedly lower plasma protein binding along with a more pronounced renal elimination. Most importantly, the *C*-hydroxy analog was also less prone to in vivo deiodination, as evidenced by the accumulation of activity in the thyroid gland [7,54]. **[^123^I]2b** did not show accumulation in the U87 tumor xenografts above background signal within 24 h.

## 3. Materials and Methods

### 3.1. Reagents

Chemicals and solvents were purchased from Merck KGaA (Darmstadt, Germany), abcr GmbH (Karlsruhe, Germany), BLD Pharmatech GmbH (Reinbek, Germany), Fisher Scientific GmbH (Schwerte, Germany), and Carl Roth GmbH & Co. KG (Karlsruhe, Germany) and were used without further purification. Dry solvents were purchased from Merck KGaA in Sure/Seal™ bottles. HPLC-grade water was purified from deionized water using a Milli-Q^®^ Integral 5 system by Merck KGaA.

### 3.2. Nuclear Magnetic Resonance Spectroscopy

NMR spectra were recorded on an Agilent 400 MHz spectrometer (Agilent Technologies, Inc., Santa Clara, CA, USA) consisting of a 400/54 premium compact magnet, 400 MR DD2 console, and 400 MHz OneNMRProbe PT probe (25 °C, ^1^H NMR 399.95 MHz, ^11^B NMR 128.32 MHz, ^13^C NMR 100.58 MHz). Chemical shifts (*δ*) are reported in ppm. ^1^H NMR spectra were referenced to the solvent residual signal as internal standard for ^1^H and ^13^C: CD_3_OD (*δ*H = 3.31 ppm; *δ*C = 49.0 ppm) [127]. ^11^B chemical shifts were calculated according to the Ξ scale [128]. The ^11^B-NMR experiments were carried out with Deutero^®^ quartz NMR tubes (Deutero GmbH, Kastellaun, Germany). Spectra were processed using MestreNova (version 15.0.1−35756, Mestrelab Research, S.L.U., Santiago de Compostela, Spain). Analyses followed first order, and the following abbreviations were used throughout: s = singlet, d = doublet. Coupling constants (*J*) are given in Hz and refer to H, H-couplings.

### 3.3. High Resolution Mass Spectrometry

High-Resolution Mass spectra (HR-MS) were obtained as ESI mass spectra using a Q-TOF MS: Agilent 1260 Infinity II HPLC (Santa Clara, CA, USA; pump G7104C, autosampler G7129C, column oven G7116A, DAD detector G7117C) coupled to gamma detector Gabi Star (Elysia-raytest GmbH, Straubenhardt, Germany), followed by accurate mass Revident Q-TOF LC/Q-TOF. The measurements were performed in bypass mode using an eluent consisting of (A): CH_3_CN and (B): 0.1% formic acid in H_2_O; flow rate 0.2 mL/min. A reference mass solution containing hexakis(1H,1H,3H-tetrafluoropropoxy) phosphazene and purine was continuously co-injected via a dual AJS ESI source. The system was operated using Agilent Masshunter Workstation 3.6—LC/MS data acquisition software (Version 12.0), and data evaluation was performed using Agilent Masshunter Workstation 3.6 Qualitative Analysis software (Version 12.0 Update 1).

### 3.4. Chromatographic Methods

Thin-layer chromatography (TLC) was performed on Merck TLC Silica gel 60 F_254_ aluminum sheets (normal phase (NP), Merck KGaA, Darmstadt, Germany) or pre-coated TLC sheets ALUGRAM^®^ RP-18W/UV_254_ (reversed phase (RP), Macherey-Nagel GmbH & Co. KG, Düren, Germany) with visualization under UV (254 nm). Carborane-containing substances were stained and identified with a 5% solution of palladium (II) chloride in methanol. Radio-TLC was performed as described for TLC and visualized using CR35 Bio (Elysia-raytest GmbH, Straubenhardt, Germany). Chromatograms were analyzed using advanced image data analyzer (AIDA) software (v5.1 SP4, Raytest, Straubenhardt, Germany).

Flash chromatographic purification was performed on a Biotage^®^ Selekt System (Biotage AB, Uppsala, Sweden) using Sfär C18 D—Duo 100 Å 30 µm cartridges and the following gradient of H_2_O (A) and CH_3_CN (B): %B (span of column volumes (CV)) 11% (1 CV), 11–50% (7 CV), 50% (2 CV), 50–95% (2 CV), 95% (2 CV). 

Preparative High-Performance Liquid Chromatography (HPLC) was performed on Shimadzu LC-20A Prominence HPLC (Shimadzu Corporation, Kyoto, Japan) consisting of a degasser unit DGU-20A5R, two separate pumping units LC-A20R, sample manager SIL-20ACHT, column oven CTO-20AC, PDA-detector SPD-M20A, communication-bus module CBM-20A, fraction collector FRC-10A, and Phenomenex Jupiter Proteo C18 column (250 mm × 21.2 mm, 4 µm, 90 Å, Phenomenex Ltd., Aschaffenburg, Germany). A binary linear gradient system of 0.1% CF_3_COOH/H_2_O (A) and 0.1% CF_3_COOH/CH_3_CN (B) at a flow rate of 10 mL/min and a column temperature of 50 °C served as the eluent. LabSolutions Software V. 5.92 (Shimadzu) was used for data processing. Gradient system 1 (%B): *t*_0 min_ 60—*t*_5 min_ 60—*t*_25 min_ 95—*t*_31 min_ 95—*t*_32 min_ 60—*t*_42 min_ 60. Semi-preparative (radio) HPLC purification was performed on a Jasco HPLC system equipped with LC-NetII/ADC interface, PU-2080 Plus pump, a Ternary Gradient Unit LG-980-02, a degasser DG-980-50, a UV-2075 Plus detector (Jasco Corporation, Tokyo, Japan), a gamma detector GABI (Elysia-Raytest GmbH, Straubenhardt, Germany) and a Phenomenex Luna 10u C18(2) 100A column (250 mm × 10 mm, 10 µm, 100 Å). The purification was carried out using a binary linear gradient system of 0.1% CF_3_COOH/H_2_O (A) and 0.1% CF_3_COOH/CH_3_CN (B) at a flow rate of 4 mL/min at room temperature. Gradient system 2 (%B): *t*_0 min_ 60—*t*_5 min_ 60—*t*_38 min_ 95—*t*_39 min_ 95—*t*_45 min_ 60—*t*_50 min_ 60. Data was processed through Jasco ChromNAV Software version 2.02.05. Semi-preparative HPLC purification was performed on Merck-Hitachi Model D-7000 LaChrom comprising modules L-7100 quaternary pump, L-7450 Diode array detector, D-7000 interface unit (Merck KGaA Darmstadt, Germany; Hitachi Instruments Inc., San Jose, CA, USA), a Jasco 4-line degasser DG-2080-54, and a Phenomenex Luna 5u PFP(2) column (250 mm × 10 mm, 5 µm, 100 Å). A binary linear gradient system of 0.1% CF_3_COOH/H_2_O (A) and 0.1% CF_3_COOH/CH_3_CN (B) at a flow rate of 4 mL/min at room temperature served as the eluent. Hitachi D-7000 Chromatography Data Station Software V.4.1 was used for data processing. Gradient system 3 (%B): *t*_0 min_ 45—*t*_3 min_ 45—*t*_28 min_ 75—*t*_29 min_ 95—*t*_34 min_ 95—*t*_35 min_ 45—*t*_40 min_ 45.

UPLC−DAD-MS was performed on a Waters ACQUITY UPLC I class system (Waters Corporation, Milford, MA, USA) including an ACQUITY UPLC PDA e λ detector coupled to an Xevo TQ-S mass spectrometer and equipped with an ACQUITY™ Premier Peptide BEH C18 column (100 mm × 2.1 mm, 1.7 μm, 300 Å) along with an ACQUITY™ Premier Peptide BEH C18 VanGuard Pre-column (5 mm × 2.1 mm, 1.7 μm, 300 Å). A binary linear gradient system of 0.1% CH_3_COOH/H_2_O (A) and 0.1% CH_3_COOH in CH_3_CN/CH_3_OH (1:1, v/v, B) at a flow rate of 0.4 mL/min and a column temperature of 50 °C served as the eluent. MassLynx (v4.2 SCN986) was used for data processing. Gradient system 4 (%B): *t*_0 min_ 45—*t*_0.5 min_ 45—*t*_5.5 min_ 95—*t*_7 min_ 95—*t*_8 min_ 45—*t*_8.5 min_ 45. ESI^−^ mode was used.

HPLC-DAD was performed on a Shimadzu Nexera X2 UHPLC system (Kyoto, Japan), equipped with degassers DGU-20A3R and DGU-20A5R, pump LC-30AD, autosampler SIL-30AC, column oven CTO-20AC with two column switching valves FCV-14AH, diode array detector SPD-M30A, gamma detector GABI Star (Elysia-raytest GmbH, Straubenhardt, Germany), communication bus module CBM-20A, and a Kinetex^®^ C18 column from Phenomenex (250 mm × 4.6 mm, 5 µm, 100 Å). A binary gradient system of 0.1% *v*/*v* CF_3_COOH/H_2_O (A) and CH_3_CN (B) at a flow rate of 1 mL/min and a column temperature of 40 °C served as the eluent. Gradient system 5 (%B): *t*_0 min_ 45—*t*_10 min_ 45—*t*_11 min_ 95—*t*_16 min_ 95—*t*_17 min_ 45—*t*_25 min_ 45.

Analytical (radio-) HPLC was performed on Agilent 1200 Series (Agilent Technologies, Santa Clara, CA, USA) consisting of interface 35900E, quaternary pump G1311A, degasser G1322A, autosampler G1329A, thermostatted column compartment G1316A, and diode array detector G1315D, equipped with a gamma detector GABI (Elysia-raytest GmbH, Straubenhardt, Germany) and Purospher^®^ RP-18 endcapped (5 µm) LiChroCART^®^ 125-3 column (125 mm × 3 mm, 5 μm, 120 Å, Merck KGaA, Darmstadt, Germany). A binary gradient system of 0.1% *v*/*v* CF_3_COOH in H_2_O (A) and CH_3_CN (B) at 40 °C was used. Data was processed using OpenLAB CDS ChemStation Edition version C.01.07 SR1 (Hamburg, Germany). System 6 gradient 1 (%B): *t*_0 min_ 5—*t*_3 min_ 5—*t*_28 min_ 95—*t*_29 min_ 95—*t*_35 min_ 5—*t*_40 min_ 5, flow rate 0.75 mL/min; system 6 gradient 2 (%B) *t*_0 min_ 45—*t*_0.5 min_ 45—*t*_7 min_ 95—*t*_8.5 min_ 95—*t*_9.5 min_ 45—*t*_15 min_ 45, flow rate 0.75 mL/min; system 6 gradient 3 (%B) *t*_0 min_ 45—*t*_3 min_ 45—*t*_28 min_ 95—*t*_29 min_ 95—*t*_35 min_ 45—*t*_40 min_ 45, flow rate 0.75 mL/min.

Chemical formulas were drawn and m/z calculated using ChemDraw Professional 19.1.1.21 (PerkinElmer Inc., Waltham, MA, USA).

### 3.5. Radionuclide Production

The no carrier added [^123^I]NaI was produced in-house using a TR-Flex cyclotron (Advanced Cyclotron Systems Inc., ACSI, BC, Canada) and the gas target KIPROS 200 from ZAG Zyklotron AG (Eggenstein-Leopoldshafen, Germany) by bombardment of highly enriched [^124^Xe]xenon gas with 30 MeV protons via, among others, the nuclear reaction ^124^Xe(p,pn)^123^Xe → ^123^I. Concentration of crude [^123^I]iodide and formulation in 0.02 M aqueous NaOH was performed by ROTOP Radiopharmacy GmbH (Dresden, Germany). Aliquots containing [^123^I] iodide in an activity concentration of 20–50 MBq/µL were used for further experiments and diluted accordingly with 0.02 M NaOH.

### 3.6. Synthesis of Reference Compounds

**2a/2b** Sodium 9/11-iodo-7-{[5-methoxy-3-(2-methoxy-2-oxoethyl)-2-methyl-1*H*-indole-1-yl]carbo-nyl}-nonahydrido-(10,11)/(9,10)-µ-hydrido-7,8-dicarba-*nido*-undecaborate(1−): 10 mg of **1** (0.025 mmol, 1 eq) were placed in the reaction vessel, dissolved in 10 mL MeOH, followed by addition of 85.7 µL of the peracetic acid 40% (0.500 mmol, 20 eq) at room temperature and 250 µL of NaI (0.025 mmol, 100 mM in MeOH, 1.0 eq). Samples of 1 µL were taken for reaction control, quenched with 15 µL of 100 mM Na_2_S_2_O_5_ in CH_3_OH, diluted to 100 µL with 45% *v*/*v* CH_3_CN/H_2_O, and analyzed using gradient system 4. After conversion was complete (2–4 h), the reaction was quenched by adding 1.5 mL of 100 mM Na_2_S_2_O_5_ in CH_3_OH (0.15 mmol, 6 eq). The solvent was evaporated under reduced pressure, the crude product dissolved in 11% *v*/*v* CH_3_CN/H_2_O, and inorganic salts were removed using flash chromatography as described. The fractions containing a mixture of **2a** and **2b** were united, diluted with water and lyophilized. Further purification was performed using system 3. After solvent removal by solid phase extraction and evaporation of EtOH under reduced pressure, **2a** (0.54 mg, 4%) and **2b** (1.96 mg, 15%) were obtained as pale-yellow powders. Further, by this method, degradation products **3a** and **3b** (sodium 9/11-iodo-7-{[5-methoxy-3-(carboxymethyl)-2-methyl-1*H*-indole-1-yl]carbonyl}-nonahydrido-(10,11)/(9,10)-µ-hydrido-7,8-dicarba-*nido*-undecaborate(1−)) were obtained in trace amounts (not weighable) as off-white powders.

Analytical data for **2a**: HR-ESI-MS (negative mode) *m*/*z* [M-Na]^−^ calculated for C_16_H_24_B_9_INO_4_^−^ 519.1629; found 519.1634; the observed isotopic pattern was in accordance with the calculated one. HPLC *t*_R_ = 12.2 min, purity in isolated HPLC fraction 98.9% (system 6 gradient 3).

Analytical data for **2b**: ^1^H NMR (400 MHz, CD_3_OD): δ = 7.12 (d, ^3^*J*_H,H_ = 8.8 Hz, 1 H, C*H*_indole-C7_), 6.92 (d, ^4^*J*_H,H_ = 2.4 Hz, 1 H, C*H*_indole-C4_), 6.67 (dd, ^3^*J*_H,H_ = 8.7, ^4^*J*_H,H_ = 2.4 Hz, 1 H, C*H*_indole-C6_), 3.80 (s, 3 H, OC*H*_3_), 3.66 (s, 2 H, C*H*_2_), 3.66 (s, 3 H, COOC*H*_3_), 2.86 (s, 1 H, C*H*_carborane_), 2.35 (s, 3 H, C*H*_3_), 3.1–0.0 (br, 8 H, B_8_*H*_8_), −2.80 (br, 1 H, bridging *H*). Signals at δ = 7.25 (d, *J* = 9.0 Hz, 1 H), 6.94 (d, *J* = 2.6 Hz, 2 H) at a molar ratio of 0.14 were observed and assigned to residual amounts of **2a**. Other signals of **2a** were superimposed by the signals of **2b**. ^11^B{^1^H} NMR (128 MHz, CDOD) δ = −5.3 (s, 1 B), −8.8 (s, 1 B), −11.9 (s, 1 B), −13.4 (s, 1 B), −15.1 (s, 1 B), −20.7 (br, 2 B), −34.4 (br, 1 B), −40.6 (s, 1 B). ^13^C NMR signals from HSQC (400 MHz/100 MHz, CD_3_OD): δ = 111.5 (indole-C_7_), 110.9 (indole-C_6_), 101.0 (indole-C_4_), 56.3 (O*C*H_3_), 55.3 (*C*H_carborane_), 52.1 (COO*C*H_3_), 30.4 (*C*H_2_), 11.1 (*C*H_3_); signals of quaternary carbon atoms (indole-C_2_, indole-C_3_, indole-C_3a_, indole-C_5_, indole-C_7a_, and carbonyl groups) could not be resolved in the HSQC. HR-ESI-MS (negative mode) m/z [M−Na]^−^ calculated for C_16_H_24_B_9_INO_4_^−^ 519.1629; found 519.1630; the observed isotopic pattern was in accordance with the calculated one. HPLC *t*_R_ = 12.5 min, purity in isolated HPLC fraction 99.1% (system 6 gradient 3) as used for identification of **[^123^I]2b**, purity in NMR sample 84.1% (system 6 gradient 3).

Analytical data for **3a**: ESI-MS (negative mode, system 4) *m*/*z* [M−Na]^−^ calculated for C_15_H_22_B_9_INO_4_^−^ 505.1472; found 505.1479, the observed isotopic pattern was in accordance with the calculated one. HPLC *t*_R_ = 8.7 min, purity in isolated HPLC fraction 58.3% (system 6 gradient 3).

Analytical data for **3b**: ESI-MS (negative mode, system 4) *m*/*z* [M−Na]^−^ calculated for C_15_H_22_B_9_INO_4_^−^ 505.1472; found 505.1478; the observed isotopic pattern was in accordance with the calculated one. HPLC *t*_R_ = 9.3 min, purity in isolated HPLC fraction 92.2% (system 6 gradient 3).

### 3.7. COX Inhibition Assay

The COX inhibition activity against ovine COX-1 and human recombinant COX-2 was determined using the COX Fluorescent Inhibitor Screening Assay Kit (Cayman Chemical Company, Ann Arbor, MI, USA) according to the manufacturer’s instructions as reported [129]. The compounds were screened at a concentration of 100 µM in duplicate. For determination of the *IC_50_* values, compounds were assayed in a concentration range from 0.32 to 320 µM in duplicate. *IC_50_* values were determined with GraphPad Prism 10 (v10.4.2) by fitting to the equation y = A_2_ + (A_1_ − A_2_)/(1 + (x/x_0_)^p^) and are given as absolute *IC_50_* values. COX-2 selective inhibitor celecoxib and COX-1 selective inhibitor SC-560 served as reference compounds (Table 1).

### 3.8. Radiosynthesis of [^123^I]2b

10 µL of precursor **1** in a 10 mM stock solution in DMSO was mixed with 15 µL of water and 15 µL of 0.02 M aqueous H_3_PO_4_. 10 µL of 10 mM aqueous NCS solution was added, followed by 60–530 MBq [^123^I]NaI in 20 µL of 0.02 M aqueous NaOH. After 10 min, the reaction was quenched by adding 20 µL of 100 mM aqueous Na_2_S_2_O_5_. For higher product activity amounts, up to 8 reactions were performed in parallel and united after quenching. The crude product was diluted with 50% *v*/*v* CH_3_CN/H_2_O to a total volume of 1.8 mL and purified using system 2. The collected fraction was diluted with 20 mL H_2_O and passed through a CHROMAFIX^®^ C_18_ ec (S) solid phase extraction (SPE) cartridge (Macherey-Nagel GmbH & Co. KG, Düren, Germany). The cartridge was rinsed with 5 mL H_2_O and dried for 3 min, applying vacuum and drawing air through it before elution with 1 mL EtOH. The obtained solution was concentrated at 70 °C under reduced pressure and a gentle stream of nitrogen gas. Starting and product activity was determined using an ISOMED 2000 activimeter (MED Nuklear-Medizintechnik Dresden GmbH, Dresden, Germany) to calculate activity yield and RCY. RCP and *A*_m_ were determined using radio-HPLC (system 6 gradients 2 and 3).

### 3.9. logD_7.4_ Determination

logD_7.4_ was determined with the shaking flask method. The SPE cartridge with the tracer was eluted with 1 mL of organic phase (*n*-octanol saturated with PBS pH 7.4). Aliquots of 200 µL were diluted to 750 µL and added to an equal volume of aqueous phase (PBS pH 7.4 saturated with *n*-octanol). The mixture was vortexed vigorously for 30 s and centrifuged at 16,100× *g* for 5 min at 20 °C. 500 µL of the organic phase was transferred to another vial containing the same amount of aqueous phase. The mixture was vortexed and centrifuged as before, and the activity of aliquots of both phases was measured using a well-type counter (ISOMED 2100, NUVIA Instruments GmbH, Dresden, Germany). logD_7.4_ was determined from the decay-corrected activities: logD_7.4_ = log(*A*_*n*-oct_/*A*_PBS_).

### 3.10. Plasma Separation from Whole Blood

For human lithium heparin plasma, venous blood (4.5 mL) from one healthy, male volunteer who was not fasting or on any medication was collected into Vacuette^®^ LH Lithium Heparin plasma separator tubes (Greiner Bio-One GmbH, Frickenhausen, Germany). The tubes were allowed to stand on ice for 30 min, protected from light, followed by centrifugation at 2000× *g* for 15 min at 20 °C. Samples were visually checked for hemolysis and interference; the plasma layer was subsequently frozen in liquid nitrogen and lyophilized. The resulting powder was stored at 4 °C (protected from light) and reconstituted by adding the appropriate amount of water prior to use.

### 3.11. Ultrafiltration Assay

An ultrafiltration assay was used to determine the binding of the iodine-123-labeled compound to plasma proteins. Amicon^®^ Ultra-0.5 centrifugal filters (MWCO 10 kDa, Merck KGaA, Darmstadt, Germany) were used to separate the free fraction of the radioligand from the protein-bound fraction as previously described by us [130]. The radioligand (approx. 75 kBq) in a volume of 4 μL EtOH was added to 796 µL of plasma or PBS and mixed by repeated pipetting. Aliquots (250 μL) were loaded into the centrifugal filters and centrifuged at 14,000× *g* at 20 °C for 30 min. After centrifugation, the filter and the bottom cup with the collected filtrate were each measured in a well-type counter (ISOMED 2100, NUVIA Instruments GmbH, Dresden, Germany).

### 3.12. Stability Studies in Vitro

In vitro stability of **[^123^I]2b** was analyzed after incubation of the radiotracer with 0.9% saline solution, PBS and plasma. 10–25 MBq of the radiotracer in 20 µL EtOH was added to 380 µL of the respective medium and incubated at 37 °C (plasma) and room temperature (0.9% saline solution, PBS), respectively. Samples of 40 µL were withdrawn at distinct time points and either analyzed directly (0.9% saline solution, PBS) or after protein precipitation (plasma). To this end, the aliquot of 40 µL was added to 160 µL of ice-cold CH_3_CN. The mixture was vortexed for 30 s, stored on ice for 4 min, and centrifuged (5 min at 14,000× *g*, 4 °C). Aliquots for analytical radio-HPLC (100 µL, system 6, gradient 3) and radio-TLC (2 µL, RP 60% *v*/*v* CH_3_CN/H_2_O) were withdrawn from the supernatant.

Liver microsome experiments with **[^123^I]2b** in the presence of NADPH (oxidizing conditions) were performed using Mouse (CD-1) Microsomes (Gibco™, Cat. No. MSMCPL, Lot MS053-A, Thermo Fisher Scientific Inc., Waltham, MA, USA) according to the procedure described by us with some modifications [131]. Incubations had a final volume of 250 µL. The radiotracer dissolved in ethanol (2 µL; 2 MBq/µL) was diluted with PBS (398 µL; 0.5% *v*/*v* ethanol; 0.2% *v*/*v* final). PBS (112.5 µL), mouse liver microsomes (12.5 µL of 20 mg/mL stock; 1 mg/mL final), and the radiotracer solution (100 µL) were mixed in a 1.5 mL Eppendorf tube and preincubated at 37 °C for 5 min. Subsequently, NADPH (25 µL of a freshly prepared 20 mM solution in PBS, 2 mM final) was added, and the mixture was further incubated at 37 °C. After distinct time points (5, 10, 15, and 30 min), an aliquot (40 µL) was withdrawn and subjected to protein precipitation as described before. Samples were analyzed using radio-TLC (RP, 0.1% TFA in 60% *v*/*v* CH_3_CN/H_2_O). Testosterone (40 µM final concentration) in place of the radiotracer was used as a positive control for oxidation. Complete conversion of testosterone was confirmed by HPLC-DAD (system 5) after 60 min.

### 3.13. Cell Uptake Studies

The human glioblastoma cell line U87 MG (# HTB-14) was purchased from ATCC (Manassas, VA, USA). The human glioblastoma cell line U251 MG (U251) (09063001) was purchased from ECACC (Salisbury, UK). Knockout of COX-2 in U87 (U87^COX−2KO^) cells using CRISPR/Cas9 technology was performed as described previously [98]. All cells were cultured in Dulbecco’s modified Eagle’s medium supplemented with 10% *v*/*v* fetal calf serum (FCS) and 1 U/mL penicillin/streptomycin (all reagents from Biochrom, Berlin, Germany) under normoxic conditions (37 °C, 5% CO_2_).

Radiotracer uptake studies were performed in confluent monolayers as described elsewhere with some modifications [11,132]. In brief, cells were seeded in 24-well plates at a density of 5 × 10^4^ cells/mL and grown to approximately 80% confluence. A solution of **[^123^I]2b** was added to the cells (200 µL per well, 0.10–0.12 MBq/mL, <0.1% *v*/*v* EtOH content) and cellular binding and uptake were investigated after the indicated time points at 37 °C. For blocking experiments, cells were pre-incubated for 30 min with the respective blocking agent at a 100 µM final concentration before addition of the radiotracer. The use of DMSO stock solutions resulted in a residual concentration of 1% *v*/*v* DMSO content. Tracer uptake was stopped by the addition of 1 mL ice-cold PBS and removal of incubation media, followed by washing the cells three times with PBS and final addition of 0.5 mL NaOH (0.1 M containing 1% *w*/*v* sodium dodecyl sulfate) for cell lysis. The radioactivity in the cell extracts was measured with a Wizard^2®^ 2480 automatic gamma counter (PerkinElmer Inc., Waltham, MA, USA). Total protein concentration in the samples was determined by the bicinchoninic acid method as described before [133]. Uptake data are expressed as percent initial dose per mg protein (%ID/mg protein).

### 3.14. Flow Cytometry

For flow cytometry analysis, approx. 10^6^ U87 cells per well (0.33 × 10^6^ cells/mL) were seeded in 6-well plates and cultured for 24 h, as described. Cell culture medium was removed, and 1.5 mL of fresh medium (untreated cells) or medium containing **1** in concentrations between 0 and 100 µM was added (containing 1% *v*/*v* DMSO). After a 30 min period of incubation at 37 °C, the medium was removed, and 1.5 mL of fresh medium (unstained cells) or medium containing 1 µg/mL propidium iodide (PI) was added. Cells were further incubated for 5 min at room temperature (20 °C) under light exclusion. Cells were then carefully rinsed with 1.5 mL PBS and dissociated from the dish by exposure to 200 µL of 2 mM EDTA in PBS at 37 °C for 5–10 min. Subsequently, 1 mL of FACS buffer (PBS containing 2% *m*/*v* heat-inactivated fetal bovine serum, 0.01% *m*/*v* NaN_3_ and 5 mM EDTA) was added, and samples were stored on ice until analysis with an Attune NxT Acoustic Focusing Cytometer (Invitrogen, Thermo Fisher Scientific Inc., Waltham, MA, USA). For the detection, the yellow laser with an excitation of 561 nm and the emission filter YL2 (620/15 nm) was used. Detector settings were optimized using unstained cells. The forward and side scatter parameters were adjusted to appropriately gate single cells. A volume of 50 μL per sample was analyzed at a flow rate of 500 μL/min, resulting in approx. 30,000 analyzed events per sample (singlets).

### 3.15. Calcein Efflux

For the calcein efflux assay, approx. 3.3 × 10^5^ U87 cells per well (1.65 × 10^5^ cells/mL) were seeded in a 96-well plate and cultured for 24 h, as described. Cell culture medium was replaced with 100 µL of fresh medium, followed by the addition of 50 µL of medium containing compound **1** (4% *v*/*v* DMSO). After 15 min of incubation at 37 °C, 50 µL of medium containing calcein-AM was added (final concentration 0.25 µM; 2% *v*/*v* DMSO, final DMSO concentration 1.5% *v*/*v*). After an additional 15 min of incubation at 37 °C, the medium was removed, and cells were washed twice with fresh medium before addition of 200 µL of fresh medium. Calcein fluorescence was measured immediately using a Cytation^TM^ 5 imaging reader (BioTek Instruments GmbH, Bad Friedrichshall, Germany) with excitation at 496 nm and emission at 517 nm. Washing and fluorescence measurements were repeated after 165 min of incubation at 37 °C. Fluorescence values were corrected for background fluorescence measured in the absence of calcein and normalized to initial fluorescence for each concentration.

### 3.16. Tumor Xenograft Model

All animal experiments were carried out according to the guidelines of the German Regulations for Animal Welfare and have been approved by the local Ethical Committee for Animal Experiments (reference number: DD24.1-5131/499/49). General anesthesia was induced and maintained by inhalation of 10% (*v*/*v*) desflurane in 30/70 (*v*/*v*) oxygen/air. Animals were warmed during anesthesia at 37 °C. Female Rj:NMRI-*Foxn1*^*nu*/*nu*^ mice (Janvier Labs S.A.S., Le Genest-Saint-Isle, France) were xenotransplanted by subcutaneous injection with 5 × 10^6^ for U87 glioblastoma cells suspended in 100 µL Dulbecco’s PBS. The animals’ general condition was monitored daily, and their weight and tumor growth were measured and recorded two to three times a week. Tumor size was determined by caliper, and tumor volume was calculated using the formula *V* = π/6 × *abc*, assuming a triaxial ellipsoid with the axes *a*, *b*, and *c*.

For urine collection, animals were allowed to roam separately in an empty, clean, conventional cage. After spontaneous miction, the urine was aspirated with a pipette tip and immediately used for further analysis. After the final experiment, anesthetized animals were sacrificed via cervical dislocation.

### 3.17. Quantitative SPECT Imaging

Quantitative SPECT imaging and extraction of tissue-specific uptake values were performed as described previously [134], with some modifications. In brief, for extracting the region-averaged standardized uptake values (SUVmean) from the heart’s blood content, three-dimensional regions of interest (ROIs) were generated within spherical preselection masks including voxels with intensities above thresholds (% of maximum voxel intensity) of >40%. Thresholds were manually adjusted to provide an ROI volume of 30 mm^3^. Time courses of the SUVmean from the heart’s blood content were analyzed using the non-linear regression model ‘two-phase exponential decay’, as implemented in Prism 11 (GraphPad Software Inc., Boston, MA, USA), with ‘initial’ and ‘plateau’ values constrained to 13.2 and 0, respectively. The initial SUVmean in the blood was calculated from the radioligand dose administered to the mice’s total blood volume at the time point of injection. The total blood volume of mice was estimated using the following body weight-based calculation formula, which has been reported elsewhere [135]: *V*_blood_ [mL] = 0.074 [mL/g] × body weight [g].

### 3.18. Metabolite Analysis in Urine

To test in vivo stability, urine of a female U87 xenografted NMRI^*nu*/*nu*^ mouse (*n* = 1, body weight 30.4 g, injected activity of **[^123^I]2b** 9.7 MBq) was collected 1 h after tracer injection. In total, 10 µL of urine sample was mixed with 20 µL of 15% *v*/*v* trichloroacetic acid (TCA) in water, vortexed for 10 s and centrifuged (5 min, 14,000× *g*, 4 °C). A sample for radio-TLC was withdrawn from the clear supernatant (1 µL) and analyzed on RP-TLC using 0.1% TFA in 50% *v*/*v* CH_3_CN/H_2_O.

## 4. Conclusions

The *nido*-carborane–based COX-2 inhibitor **1** was employed for radioiodination, followed by in vitro and in vivo studies. Electrophilic iodination of **1** was shown to be feasible, yielding the regioisomers **2a** and **2b**. The iodinated compounds exhibited lower COX-2 potency and selectivity than **1**, with **2b** being the more potent isomer selected for radiolabeling. Radiosynthesis, purification, and formulation of **[^123^I]2b** were achieved successfully but with a low RCY. The radiotracer exhibited high stability in formulation solvents like EtOH, 0.9% saline solution and PBS, an adequate lipophilicity, but also high protein binding as well as limited stability in human plasma or murine liver microsome incubations. A contribution of COX-2 in cell uptake of **[^123^I]2b** was supported by in vitro studies, but observations of increased uptake mitigated by *nido*-carboranes **1** and **2b** raised further questions regarding the underlying mechanism that could not be elucidated in this study. In a xenograft model, balanced renal and hepatobiliary excretion and marked thyroid uptake of activity, but no tumor accumulation of **[^123^I]2b** was observed over 24 h. Consequently, despite the promising track of the *nido*-indoborin scaffold, **[^123^I]2b** is not suited as a COX-2 radiotracer. A future strategy might involve the connection to an internalization unit, such as the integrin-targeting RGD peptide sequence, to enhance tumor targeting [136]. The B–I bond demonstrated better in vitro and in vivo stability compared to the thiophene C–I bond of a recently investigated radioiodinated COX-2/5-LO targeted tracer [134], but was not completely resistant to oxidative degradation in vivo, as evidenced by thyroidal accumulation and renal elimination of free [^123^I]iodide.

Generally, the use of iodinated carboranes as radiotracers remains a promising strategy regarding the stability of the B-I bond and the variety of radioisotopes of iodine that are available and viable for radiopharmaceutical and nuclear medicine purposes (^123^I, ^124^I, ^125^I, ^131^I). The inherent challenges of this technique using electrophilic iodination are the creation of regioisomers, complicating purification and potentially impairing target binding. Isotopic exchange, on the other hand, leads to inferior molar activities. We encourage the use of indirect labeling approaches instead, e.g., via activated esters [8,59,137]. However, this technique is limited to antibodies, peptide-based ligands or small molecules with a substantially large spacer unit to avoid interference between the labeling moiety and the pharmacophore.

## Figures and Tables

**Table 1:** COX Inhibition of compounds **1**, **2a**, and **2b** using ovine COX-1 and human recombinant COX-2 with SC-560 and celecoxib as references was determined in a COX fluorescent inhibitor screening assay. % Inhibition was determined by screening in duplicate at 100 µM.

Compound	% Inhibition at 100 µM		*IC*_50_ in µM		SI ^a^
	COX-1	COX-2	COX-1	COX-2	
**1** ^b^	/^c^	/	>100	0.91	>109
**2a** ^d^	52	66	10.6	42.1	0.25
**2b** ^e^	76	90	15.8	4.42	3.6
**SC-560** ^f^	/	/	0.006 ± 0.002	/	/
**Celecoxib** ^f^	/	/	/	0.13 ± 0.06	/

^a^ COX-2 selectivity index, SI = *IC*_50_(COX-1)/*IC*_50_(COX-2). ^b^ reported earlier by us [69]. ^c^ / = not determined. ^d^ purity 75% (HPLC, 254 nm). ^e^ purity 91% (HPLC, 254 nm) ^f^ *IC*_50_ given as mean ± SD (*n* = 2).

**Table 2:** Optimization of the radioiodination parameters (A–C oxidant, D–F solvent, G–K temperature, L–O reaction time, Q–S pH adjusting additive). Conditions marked in bold were chosen for further optimization steps. Each entry A–S represents an independent experiment.

	Oxidant	Solvent	Temperature	Reaction Time	Additive	RCC in % (HPLC)	
						Total	[^123^I]2b
A	CAT	H_2_O	20 °C	10 min	None	34	20
B	**NCS**	H_2_O	20 °C	10 min	None	**58**	**23**
C	AcOOH	H_2_O	20 °C	10 min	None	7	1
D	NCS	**H_2_O**	20 °C	10 min	None	**85**	**38**
E	NCS	CH_3_CN	20 °C	10 min	None	69	8
F	NCS	DMSO	20 °C	10 min	None	17	2
G	NCS	H_2_O	**20 °C**	10 min	None	**54**	**24**
H	NCS	H_2_O	40 °C	10 min	None	74	30
I	NCS	H_2_O	70 °C	10 min	None	71	27
K	NCS	H_2_O	90 °C	10 min	None	67	24
L	NCS	H_2_O	20 °C	**10 min**	None	**33**	**13**
M	NCS	H_2_O	20 °C	60 min	None	31	10
N	NCS	H_2_O	20 °C	90 min	None	33	10
O	NCS	H_2_O	20 °C	120 min	None	35	10
P	NCS	H_2_O	20 °C	10 min	None	24	14
Q	NCS	H_2_O	20 °C	10 min	3.3 mM H_3_PO_4_	31	14
R	NCS	H_2_O	20 °C	10 min	**10 mM H_3_PO_4_**	**71**	**26**
S	NCS	H_2_O	20 °C	10 min	41.7 mM H_3_PO_4_	75	29

Activity of added [^123^I]NaI: A–K 1–5 MBq, L–O 35–37 MBq, P–S 80–83 MBq. Composition of reaction mixture: 10 µL of 10 mM precursor stock solution in DMSO, 30 µL of solvent, 10 µL of solvent containing the dissolved oxidant, 1 µL (A–O) or 10 µL (P–S) 0.02 M aqueous NaOH containing [^123^I]NaI. The oxidants were used in the following concentrations: 2 mM (CAT, NCS) and 360 mM (AcOOH).

**Table 3:** SPECT image-extracted uptake values characterizing the retention and elimination of **[^123^I]2b** in mice.

	1 h	5 h	24 h
Retention total	42.5 (36.1–48.9)	18.0 (12.5–23.5)	2.50 (1.59–3.41)
NIS-positive tissues ^a^	4.50 (1.77–7.23)	3.50 (0.77–6.23)	2.00 (0.18–3.82)
Excretion total	57.5 (51.1–63.9)	82.0 (76.5–87.5)	97.5 (96.6–98.4)
renal fraction ^b^	8.50 (2.13–14.9)	n.d.	n.d.
hepatobiliary fraction ^c^	48.5 (36.7–60.3)	n.d.	n.d.

Data presented as percent of the initially administered dose (% ID); means with 68% confidence interval; number of animals investigated *n* = 2; ^a^ major Na^+^/I^−^ symporter (NIS)-positive tissues: thyroid + salivary glands + stomach; ^b^ urinary bladder + (100%—total); ^c^ liver + gall bladder + intestine, assuming that the entire hepatobiliary fraction is still detectable inside the animal after 1 h.

## Data Availability

All data generated or analyzed during this study are included in this article and its Supplementary Information Files.

## Supplementary Materials

The following supporting information can be downloaded at: https://www.mdpi.com/article/10.3390/molecules31111944/s1, Figure S1: Reaction control iodination of **1**.; Figure S2: HPLC purification of **2a** and **2b**.; Figure S3: HR-MS-ESI of **2a**. Figure S4: HPLC purity of **2a** (system 6 gradient 3). Figure S5: ^1^H NMR of **2b** in CD_3_OD. Figure S6: HSQC NMR (400 MHz/100 MHz) of **2b** in CD_3_OD. Figure S7: ^11^B NMR (128 MHz) of **2b** in CD_3_OD. Figure S8: ^11^B{^1^H} NMR (128 MHz) of **2b** in CD_3_OD. Figure S9: Comparison of ^1^H NMR spectra of **2b** (top, blue, R = I) and **1** (bottom, red, R = H) in CD_3_OD. Figure S10: HRMS of **2b**. Figure S11: HPLC purity of **2b** (system 6 gradient 3). Figure S12. *m*/*z* (MS-ESI^−^) of **3a** (system 4). Figure S13. HRMS of **3a**. Figure S14. *m*/*z* (MS-ESI^−^) of **3b** (system 4). Figure S15: HRMS of **3a**. Figure S16: COX inhibition assay. COX-1 (left) and COX-2 inhibition (right) of **2a** (top) and **2b** (bottom). Figure S17: Optimization of radioiodination. Figure S18: **[^123^I]2b** coinjected with **2b** (system 6 gradient 3). Figure S19: Sample of **[^123^I]2b** (*t*_R_ = 12.4 min) withdrawn after 16 h of incubation with human plasma, coinjected with **3b** (*t*_R_ = 8.7 min, system 6 gradient 3). Figure S20: Possible radiometabolites of **[^123^I]2b** obtained in a murine liver microsome assay as a result of CYP and CES metabolism based on the literature-reported metabolism of indomethacin [64,65,66]. Figure S21: Radio TLC of murine liver microsome assay. Lanes from left to right: reference **[^123^I]2b**, reference **[^123^I]3b**, 60 min control. Figure S22: Cell uptake studies of **[^123^I]2b** in U87 (left column), U87^COX2KO^ (middle column), and U251 cells (right column). Figure S23: Flow cytometry analysis of U87 cells treated with different concentrations of **1**. Figure S24: Calcein efflux from U87 cells treated with different concentrations of **1**. Figure S25: Radio-TLC of urine sample collected 1.5 h after injection of **[^123^I]2b** in U87 xenografted mouse. Table S1: Batch reproducibility in the synthesis of **2b** from **1**.

## Abbreviations

The following abbreviations are used in this manuscript:

ABC	ATP-binding cassette
AcOOH	Peracetic acid
ATP	Adenosine triphosphate
BCRP	Breast cancer resistance protein
BSA	Bovine serum albumin
c.a.	Carrier added
CAT	Chloramine-T
CES	Carboxylesterase (human)
Ces	Carboxylesterase (murine)
CMC	Critical micellar concentration
COX	Cyclooxygenase
CRISPR/Cas	Clustered regularly interspaced short palindromic repeats/CRISPR associated protein
CYP	Cytochrome P450
DMSO	Dimethyl sulfoxide
eq.	Molar equivalents
EtOH	Ethanol
HPLC	High-performance liquid chromatography
HRMS	High-resolution mass spectrometry
IC	Inhibitory concentration
ID	Initial dose
MDR	Multidrug resistance
mRNA	Messenger ribonucleic acid
MRP	MDR protein
MS	Mass spectrometry
NADPH	Nicotinamide adenosyl dinucleotide phosphate
n.c.a.	No carrier added
NCS	*N*-Chlorosuccinimide
NIS	Na^+^/I^−^ symporter
NMR	Nuclear magnetic resonance spectroscopy
NSAID	Non-steroidal anti-inflammatory drug
P-gp	Permeability glycoprotein
RCC	Radiochemical conversion
RCP	Radiochemical purity
RCY	Radiochemical yield
ROI	Region of interest
SD	Standard deviation
SI	COX-2 selectivity index
SPE	Solid phase extraction
SPECT	Single-photon emission computed tomography
SUV	Standardized uptake value
TFA	Trifluoroacetic acid
TLC	Thin-layer chromatography
U(H)PLC	Ultra-high performance liquid chromatography
