# Supplementary material for: An Approach Toward Radioiodination and Radiopharmacological Evaluation of a Carborane-Containing Analog of Indomethacin
Source: Molecules. 2026 Jun 3;31(11):1944. doi: 10.3390/molecules31111944 (PMC13257756; doi:10.3390/molecules31111944)
Supplement: Supplementary file 1 [file molecules-31-01944-s001.zip › molecules-4321947-supplementary.pdf]

Radioiodination and radiopharmacological characterization of a carborane-containing analog of indomethacin

Supporting Information

Contents

List of figures 1

List of tables 2

Synthesis and purification of **2a** and **2b** 3

Analytical data for **2a** 5

Analytical data for **2b** 7

Analytical data for **3a** 12

Analytical data for **3b** 14

COX Inhibition Assay 16

Optimization of radioiodination 17

Murine liver microsome assay 19

Cell uptake studies 20

Urine analysis 21

List of figures

Figure S 1. Reaction control iodination of **1**. 3
Figure S 2. HPLC purification of **2a** and **2b**. 3
Figure S 3. HR-MS-ESI of **2a.** 5
Figure S 4. HPLC purity of **2a** (254 nm, system 6 gradient 3). 6
Figure S 5. ^{1}H NMR of **2b** in CD_3OD. 7
Figure S 6. HSQC NMR (400 MHz/ 100 MHz) of **2b** in CD_3OD. 8
Figure S 7. ^{11}B NMR (128 MHz) of **2b** in CD_3OD. 8
Figure S 8. ^{11}B{^{1}H} NMR (128 MHz) of **2b** in CD_3OD. 9
Figure S 9. Comparison of ^{1}H NMR spectra of **2b** (top, blue, R = I) and **1** (bottom, red, R = H) in CD_3OD. 9
Figure S 10. HRMS of **2b**. 10
Figure S 11. HPLC purity of **2b** (254 nm, system 6 gradient 3). 11
Figure S 12. m/z (MS-ESI^{-}) of **3a** (system 4). 12
Figure S 13. HRMS of **3a**. 13
Figure S 14. m/z (MS-ESI^{-}) of **3b** (system 4). 14
Figure S 15. HRMS of **3a**. 15
Figure S 16. COX inhibition assay. COX-1 (left) and COX-2 inhibition (right) of **2a** (top) and **2b** (bottom). 16
Figure S 17. Optimization of radioiodination. 17
Figure S 18. **[^{123}I]2b** coinjected with **2b** (system 6 gradient 3). 17
Figure S 19. Sample of **[^{123}I]2b** (t_R = 12.4 min) withdrawn after 16 h of incubation with human plasma, coinjected with **3b** (t_R = 8.7 min, system 6 gradient 3). 18

Figure S 20. Possible radiometabolites of **[^{123}I]2b** obtained in murine liver microsome assay as result of CYP and CES metabolism based on literature reported metabolism of indomethacin [64–66]....... 19
Figure S 21. Radio TLC of murine liver microsome assay. Lanes from left to right: reference **[^{123}I]2b**, reference **[^{123}I]3b**, 60 min control. 19
Figure S 22. Cell uptake studies of **[^{123}I]2b** in U87 (left column), U87^{COX2KO} (middle column), and U251 cells (right column). 20
Figure S 23. Flow cytometry analysis of U87 cells treated with different concentrations of **1**. 20
Figure S 24. Calcein efflux from U87 cells treated with different concentrations of **1**. 21
Figure S 25. Radio-TLC of urine sample collected 1.5 h after injection of **[^{123}I]2b** in U87 xenografted mouse. 21

List of tables

Table S 1. Batch reproducibility in the synthesis of **2b** from **1**........ 4

Synthesis and purification of **2a** and **2b**

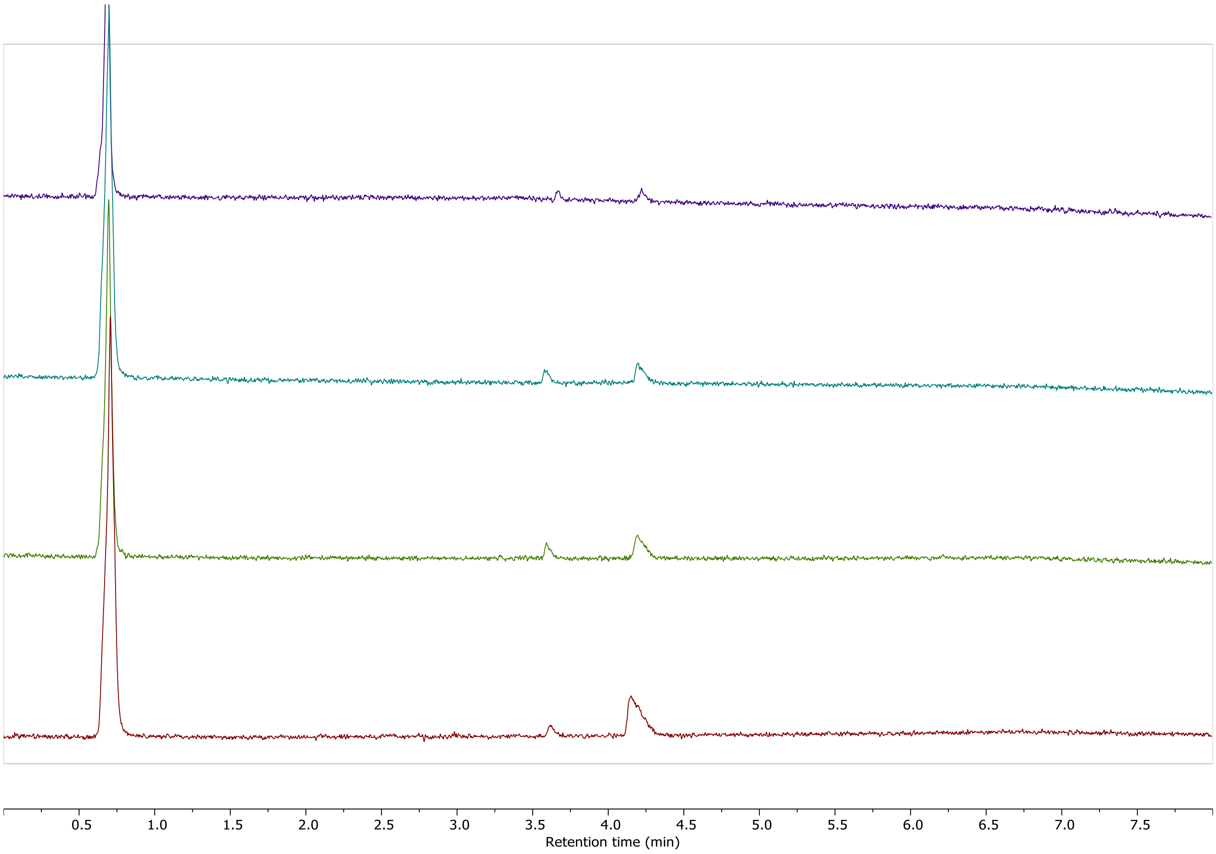

Figure S 1. Reaction control iodination of **1**.

Samples withdrawn after 10 min, 30 min, 90 min, 120 min (top to bottom). The peak at t_R 3.6 min corresponds to **1**, while the second peak at t_R 4.2 min represents a mixture of **2a** and **2b**.

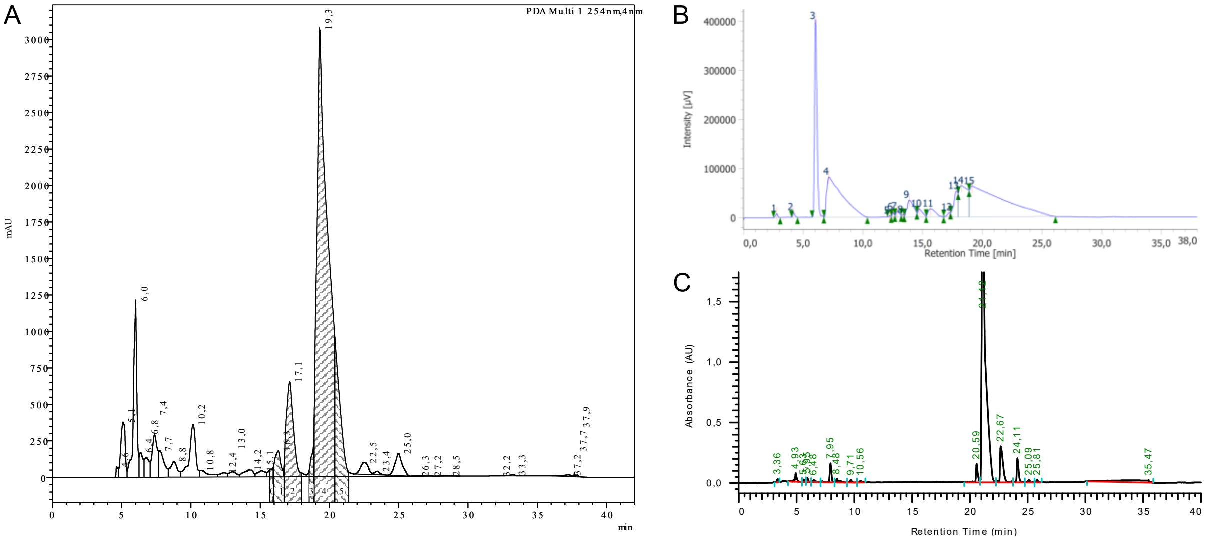

Figure S 2. HPLC purification of **2a** and **2b**.

A Attempt using system 1. **2a** and **2b** elute incompletely separated between 19 and 21 min. **B** Attempt using system 2. **2a** and **2b** elute between 18 and 26 min. **C** Successful separation of **2a** (t_R 20.6 min) and **2b** (t_R 21.1 min) using system 3.

Table S 1. Batch reproducibility in the synthesis of **2b** from **1**.

Batch #	Notes on reaction conditions	Notes on purification	Amount of **1**	Purification method(s)		Product **2b**	Yield	Purity (HPLC, 254 nm)	
				first	second			1st purification	2nd purification
55-23	DMSO stock solution of **1**	-	1.14 mg	System 1	-	Not weighable	-	100% [a,b]	-
57-23	DMSO/MeOH	-	10.4 mg	System 1	-	3.8 mg	28%	83% [a,b], 61% [c,d]	-
40-24	MeOH	-	11.3 mg	System 1	System 3	0.6 mg	4%	51–78% [c]	99% [a], 74% [c]
43-24 [e]	MeOH	-	16.4 mg	System 1	System 3	3.2 mg	15%	52% [c]	98% [a]
47-24	MeOH, different batch of 40% AcOOH	-	3.8 mg	-	-	No reaction	-	-	-
51-24	MeOH	SPE before HPLC	12.0 mg	Flash	-	2.8 mg	18%	40% [a]	-
58-24 [f]	MeOH	SPE after HPLC	3 × 10.0 mg [g]	System 1	System 2	10.5 mg	27%	61–84% [h]	60% [a]
09-25 [i,k]	MeOH	-	2 × 8.4 mg [g]	System 3	-	n.d.	n.d.	100% [a], 38% [c]	-
12-25 [l]	MeOH	SPE after HPLC	4 × 10.5 mg [g]	Flash	System 3	8 mg	15%	85% [c]	99% [a], 86% [h]

[a] Determined immediately after purification in HPLC/ flash chromatography fraction. [b] No discrimination of isomers **2a** and **2b** due to analytical method. [c] Determined after lyophilization. [d] Storage at room temperature. [e] Batch used for HRMS of **2b**. [f] Batch used for NMR of **2b**. [g] Synthesis in the given number of batches. [h] Determined following solvent removal after SPE. [i] Batch used for HRMS of **2a**. [k] Batch used for COX assay. [l] Batch used as blocking agent in cell studies

Analytical data for **2a**

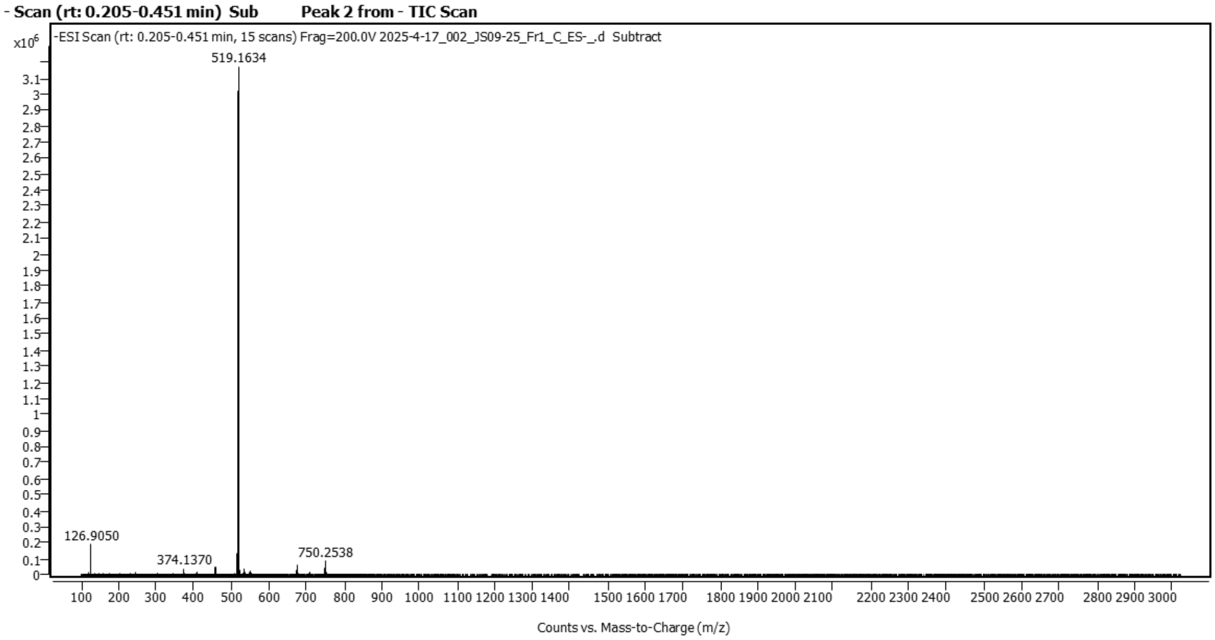

Spectrum Peaks

m/z	Z	Abund	Abund %	m/z (Calc)	Diff (ppm)	Ion Species	Formula	Ion Type
126.9050		190757	6.02					
374.1370		37347	1.18					
456.0032		27646	0.87					
456.9998		49375	1.56					
457.9964		51079	1.61					
515.1773		135636	4.28					
516.1738		565749	17.85					
517.1703		1632812	51.51					
518.1668		3017439	95.19					
519.1634	1	3170012	100.00					
520.1606	1	1407517	44.40					
521.1635	1	253313	7.99					
522.1657	1	32323	1.02					
533.1599		25111	0.79					
534.1616		33789	1.07					
535.1583		35995	1.14					
551.1534		24698	0.78					
673.0773		32138	1.01					
674.0740		57524	1.81					
675.0708	1	60467	1.91					
676.0685	1	31926	1.01					
748.2600		43600	1.38					
749.2568		79437	2.51					
750.2538	1	86351	2.72					
751.2521	1	51529	1.63					

Figure S 3. HR-MS-ESI of **2a.**

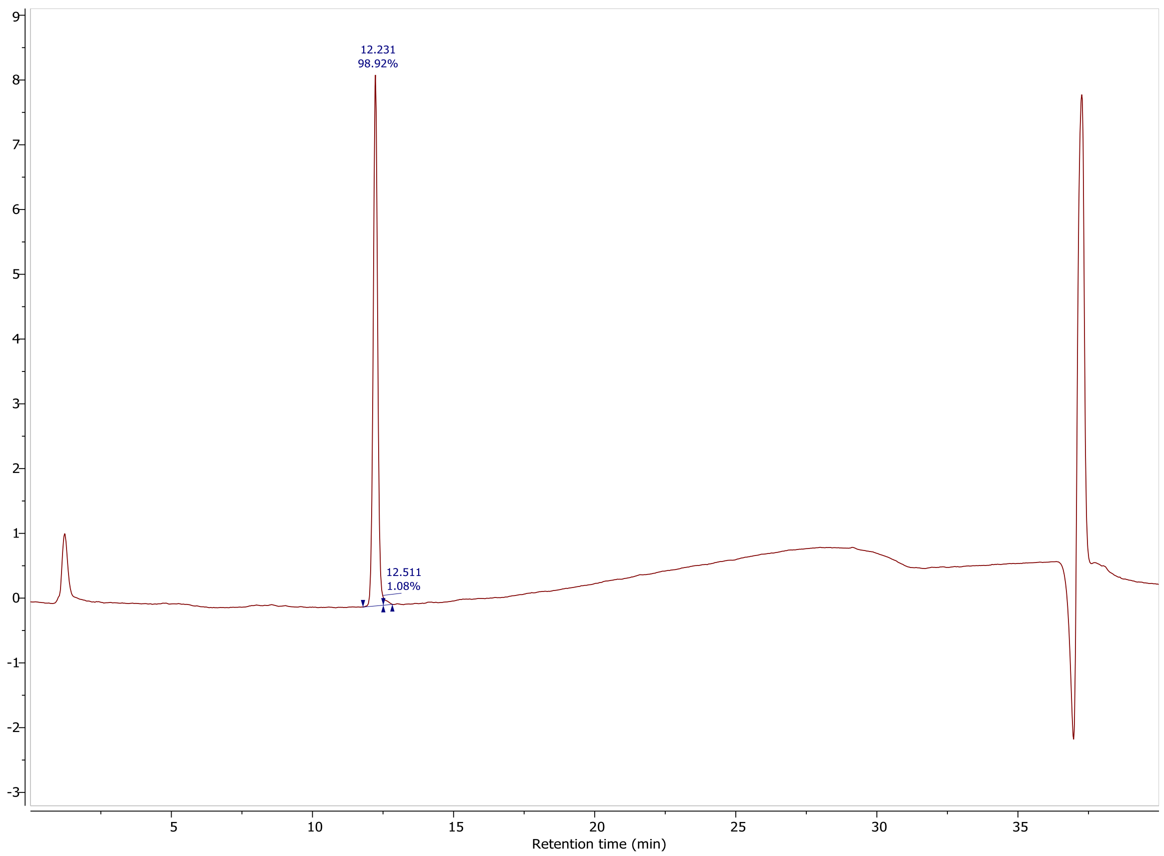

Figure S 4. HPLC purity of **2a** (254 nm, system 6 gradient 3).

Analytical data for **2b**

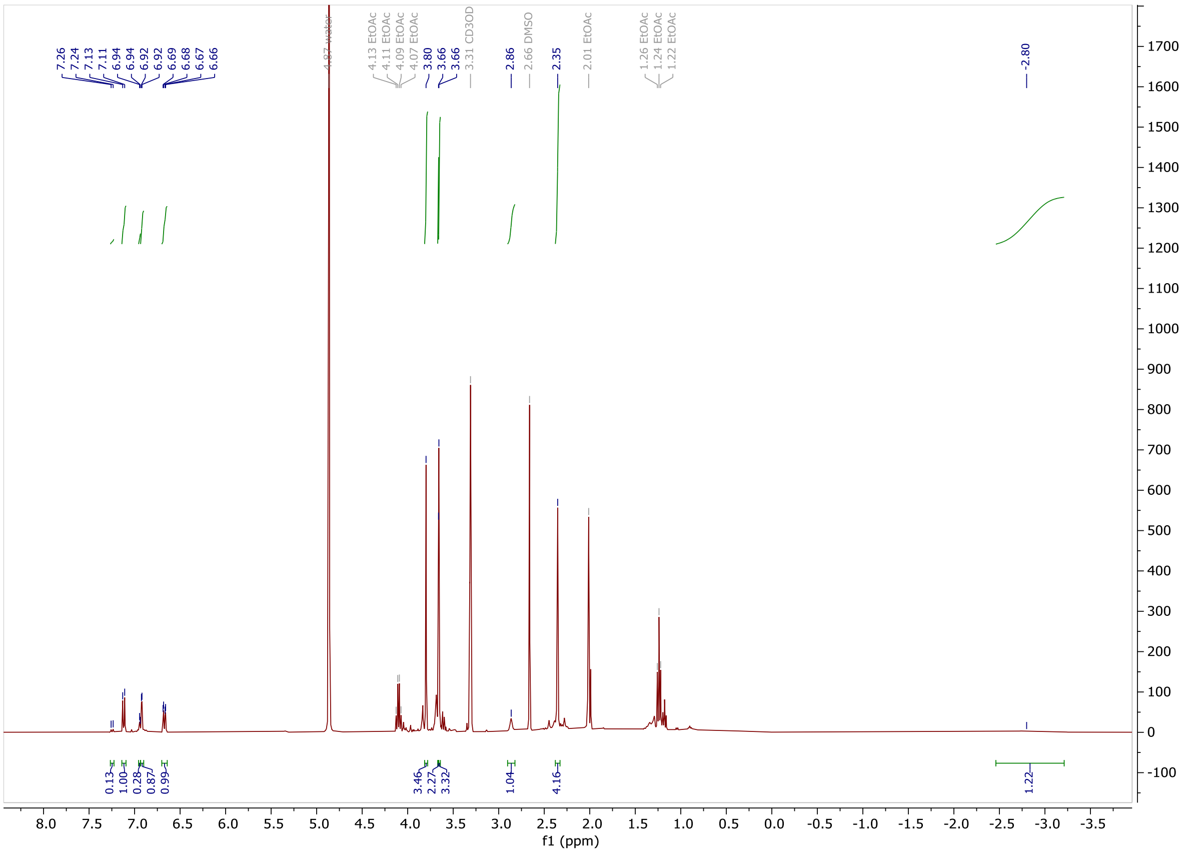

Figure S 5. ^{1}H NMR of **2b** in CD_3OD.

According to HPLC (254 nm), the sample of **2b** contained 15% **2a**. Residual signals of **2a** at a molar ratio of 0.14 are integrated between 6.9 and 7.3 ppm.

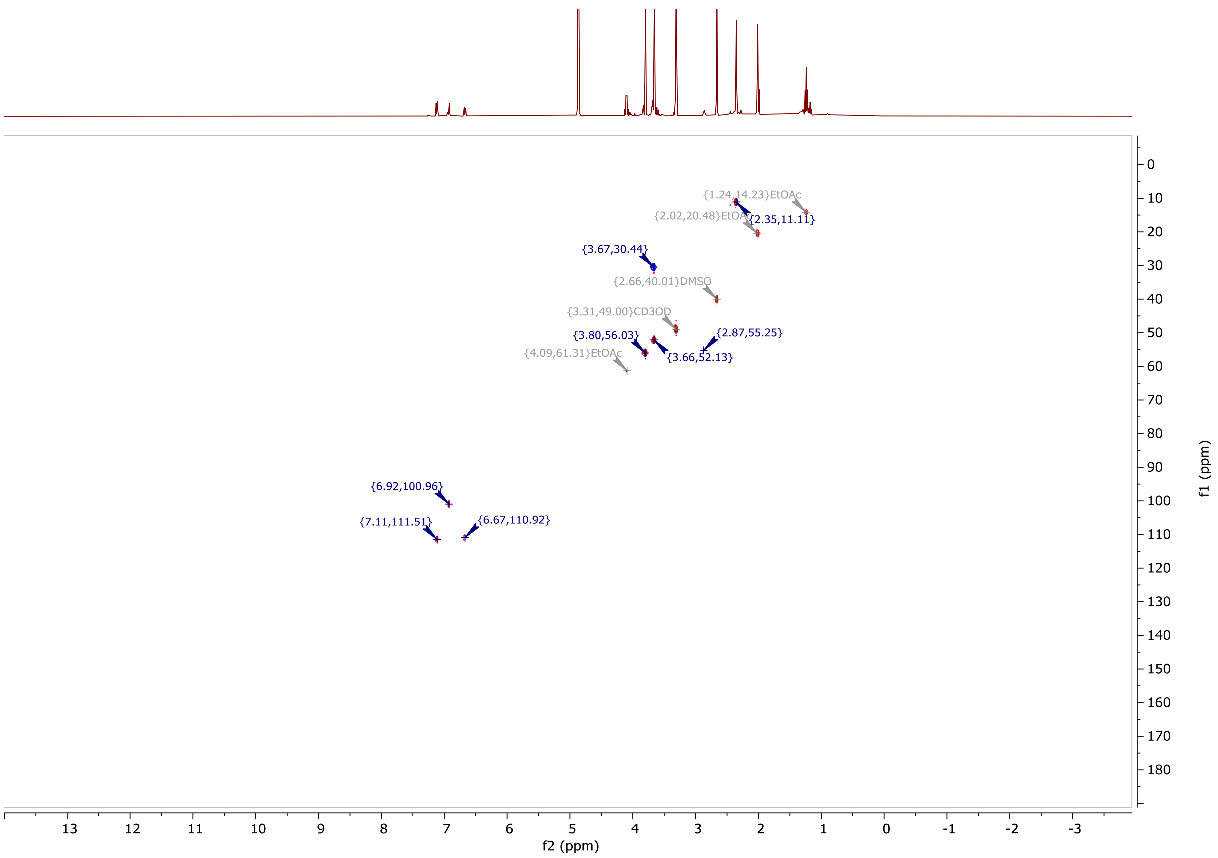

Figure S 6. HSQC NMR (400 MHz/ 100 MHz) of **2b** in CD_3OD.

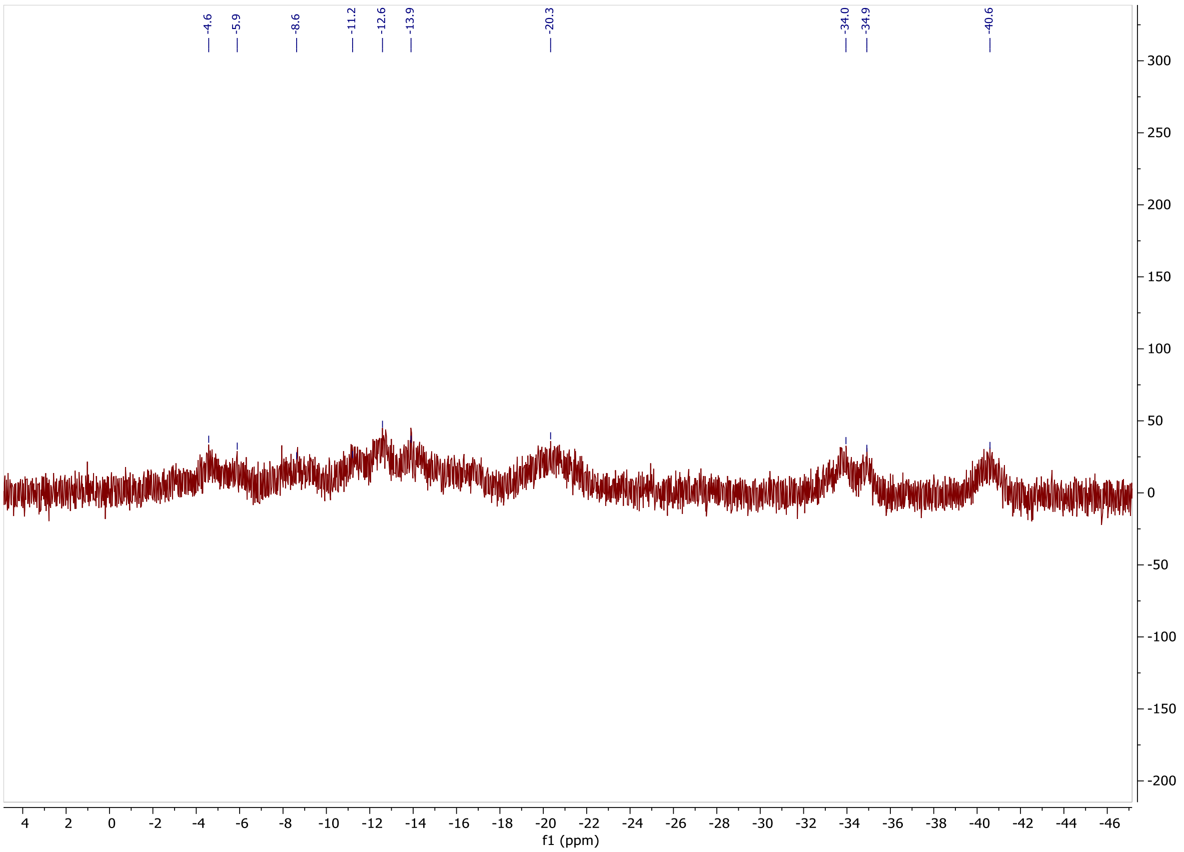

Figure S 7. ^{11}B NMR (128 MHz) of **2b** in CD_3OD.

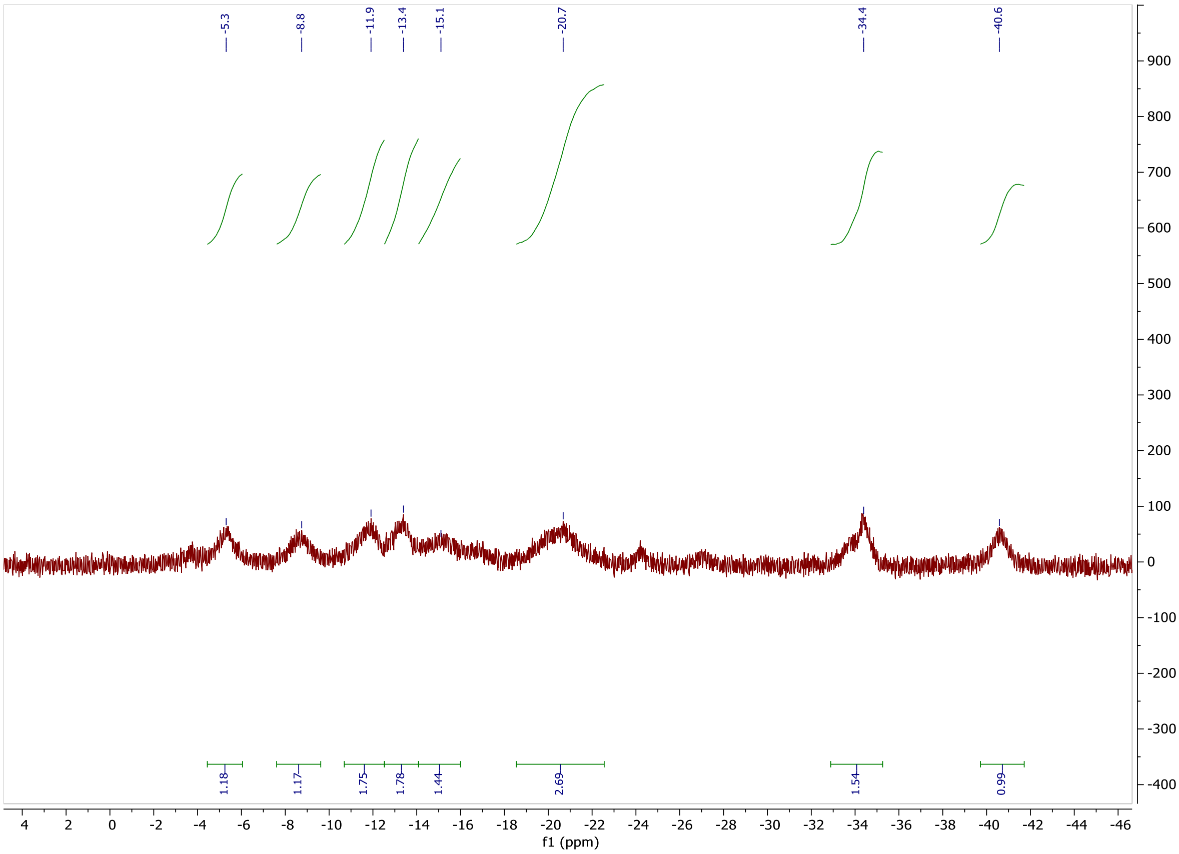

Figure S 8. $^{11}B\{^{1}H\}$ NMR (128 MHz) of **2b** in CD_3OD.

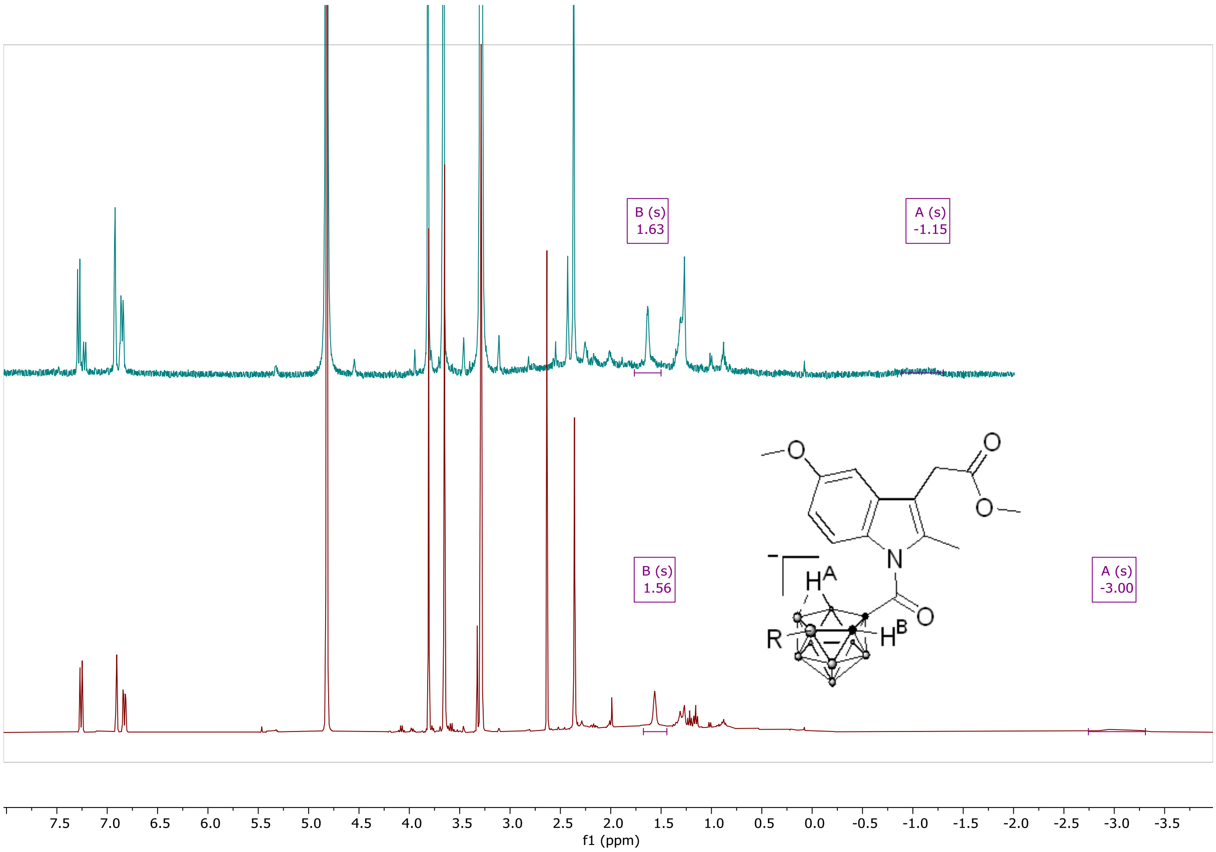

Figure S 9. Comparison of ^{1}H NMR spectra of **2b** (top, blue, R = I) and **1** (bottom, red, R = H) in CD_3OD.

Aromatic and aliphatic proton signals align, while carborane signals H^A and H^B vary in shift.

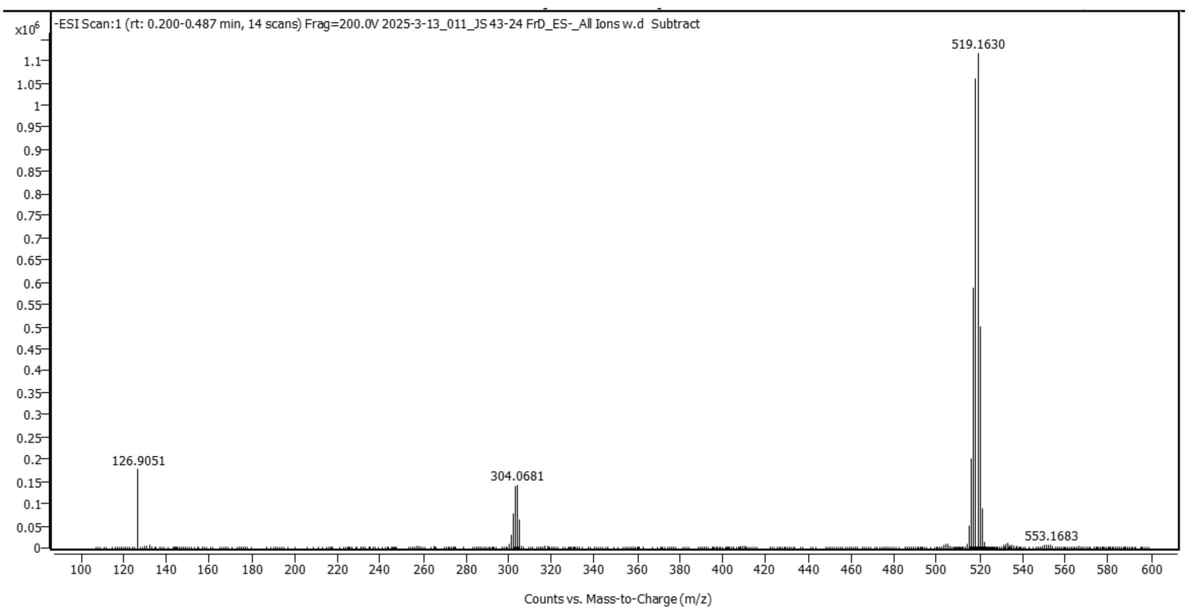

Spectrum Peaks

m/z	Z	Abund	Abund %	m/z (Calc)	Diff (ppm)	Ion Species	Formula	Ion Type
126.9051		177524	15.88					
300.0827		7371	0.66					
301.0791		27802	2.49					
302.0754		77200	6.91					
303.0718		139829	12.51					
304.0681		142377	12.74					
305.0646		64109	5.74					
504.1514		8521	0.76					
505.1472		8969	0.80					
514.1803		8357	0.75					
515.1771		49040	4.39					
516.1520		6034	0.54					
516.1735		201973	18.07					
517.1484		5791	0.52					
517.1699		586031	52.43					
518.1446		7106	0.64					
518.1664		1060271	94.85					
519.1630	1	1117840	100.00					
520.1602	1	500406	44.77					
521.1632	1	87861	7.86					
522.1655	1	12118	1.08					
532.1820		9246	0.83					
533.1782		10370	0.93					
535.1577		5887	0.53					
553.1683		5792	0.52					

Figure S 10. HRMS of **2b**.

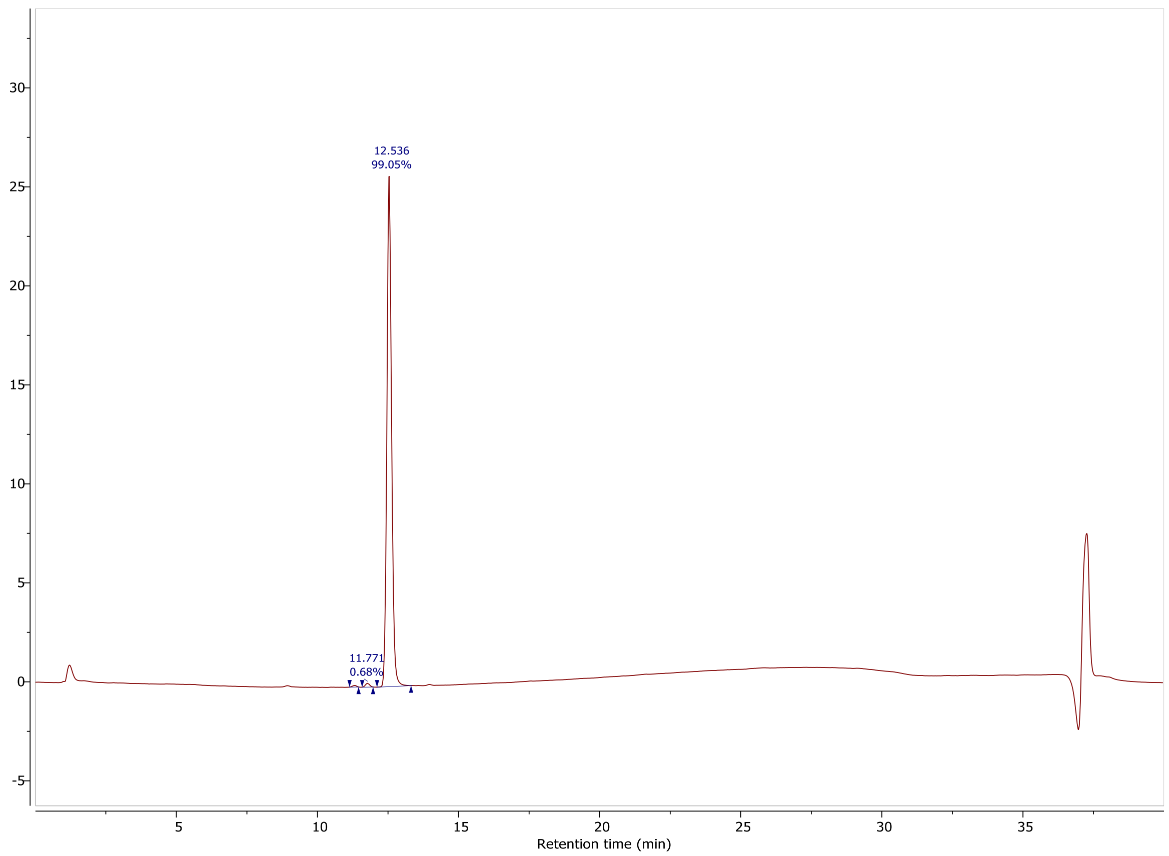

Figure S 11. HPLC purity of **2b** (254 nm, system 6 gradient 3).

Analytical data for **3a**

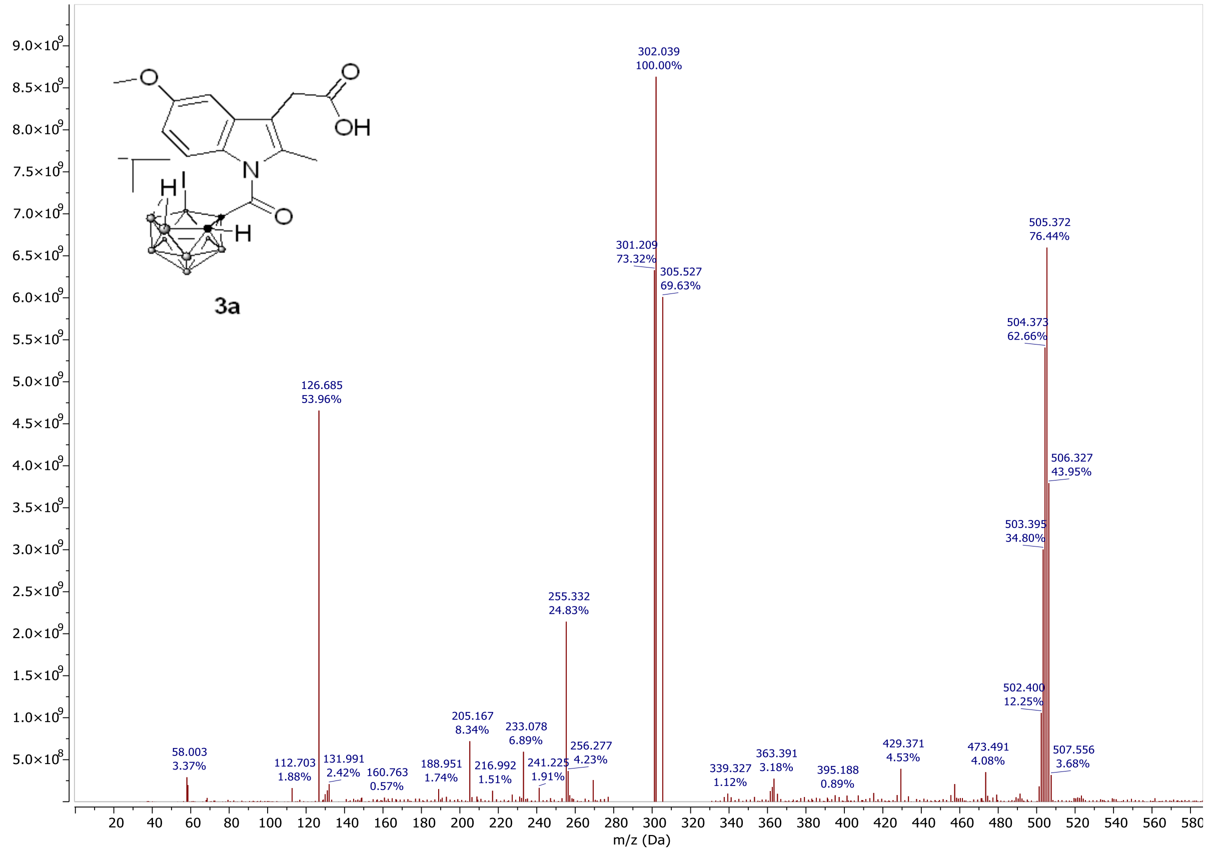

Figure S 12. m/z (MS-ESI⁻) of **3a** (system 4).

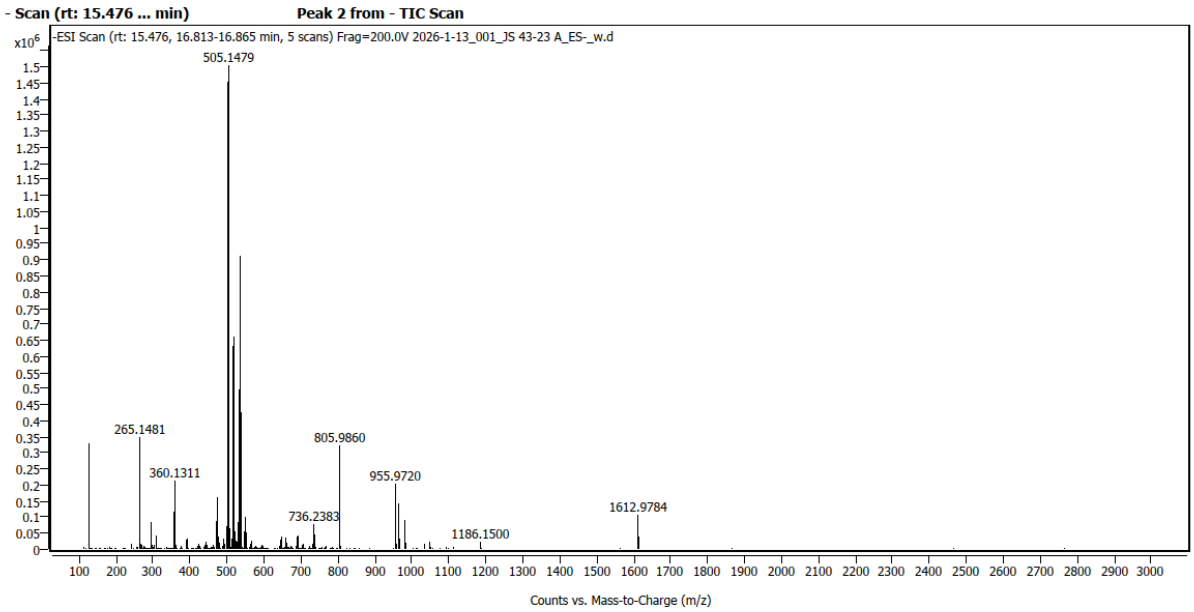

m/z	Z	Abund	Abund %	m/z	Z	Abund	Abund %	m/z	Z	Abund	Abund %
126.9051		329355	21.89					805.9860	1	320544	21.31
265.1481	1	346138	23.01	521.1637	1	56996	3.79	806.9889	1	52853	3.51
266.1513	1	44125	2.93	522.1255		48979	3.26	955.9720	1	204263	13.58
297.1531		83392	5.54	523.1220	1	53196	3.54	956.9752	1	43806	2.91
309.1742		40900	2.72	524.1191	1	24747	1.65	957.9697	1	70022	4.65
357.1419		41904	2.79	526.1330		22088	1.47	966.0007	1	141480	9.40
358.1383		116934	7.77	527.1299		22328	1.48	967.0037	1	31683	2.11
359.1347		204612	13.60	530.9671		84308	5.60	982.9908	1	91498	6.08
360.1311	1	212866	14.15	532.1829		32449	2.16	983.9939	1	19359	1.29
361.1275	1	97831	6.50	533.1640		29784	1.98	1050.0221		21895	1.46
392.2700		29405	1.95	533.1854		50210	3.34	1186.1500		22961	1.53
393.2667		30940	2.06	534.1615		49551	3.29	1612.9784	1	105563	7.02
442.9844		20183	1.34	534.1844		180244	11.98	1613.9815	1	39899	2.65
443.9806		21326	1.42	535.1584	1	50986	3.39				
472.1477		31905	2.12	535.1811		496435	33.00				
473.1443		88276	5.87	536.1556	1	24788	1.65				
474.1407	1	159245	10.59	536.1776		869699	57.81				
475.1373	1	162230	10.78	537.1742	1	911525	60.59				
475.1592	1	20617	1.37	538.1714	1	425231	28.27				
476.1344	1	78942	5.25	539.1744	1	82842	5.51				
476.1560	1	38555	2.56	548.1623		22168	1.47				
477.1527	1	38855	2.58	549.1570		55583	3.69				
478.1497	1	19508	1.30	550.1552		96695	6.43				
492.1513		31403	2.09	551.1530	1	100947	6.71				
493.1477		32837	2.18	552.1504	1	50533	3.36				
501.1621		71593	4.76	552.1720		21072	1.40				
502.1583		299230	19.89	553.1680	1	22314	1.48				
503.1548		837944	55.70	566.1516		25250	1.68				
504.1513	1	1452404	96.55	567.1483		27134	1.80				
505.1479	1	1504320	100.00	646.0432		29387	1.95				
505.1696	1	32532	2.16	647.0397		30601	2.03				
506.1449	1	687609	45.71	647.0617		20770	1.38				
506.1661	1	109238	7.26	648.0583		35616	2.37				
507.1481	1	133363	8.87	649.0550		39711	2.64				
507.1629	1	124629	8.28	660.0585		33253	2.21				
508.1582	1	65886	4.38	661.0552		34677	2.31				
515.1775		31232	2.08	691.0882		22185	1.47				
516.1740		131286	8.73	692.0848		38473	2.56				
517.1705		353566	23.50	693.0816	1	42399	2.82				
518.1670		631700	41.99	694.0790	1	21867	1.45				
519.1636	1	659508	43.84	734.2445		40806	2.71				
520.1607	1	309079	20.55	735.2413		69430	4.62				
521.1294		26910	1.79	736.2383	1	77931	5.18				
521.1424		22025	1.46	737.2364	1	45493	3.02				

Figure S 13. HRMS of **3a**.

Analytical data for **3b**

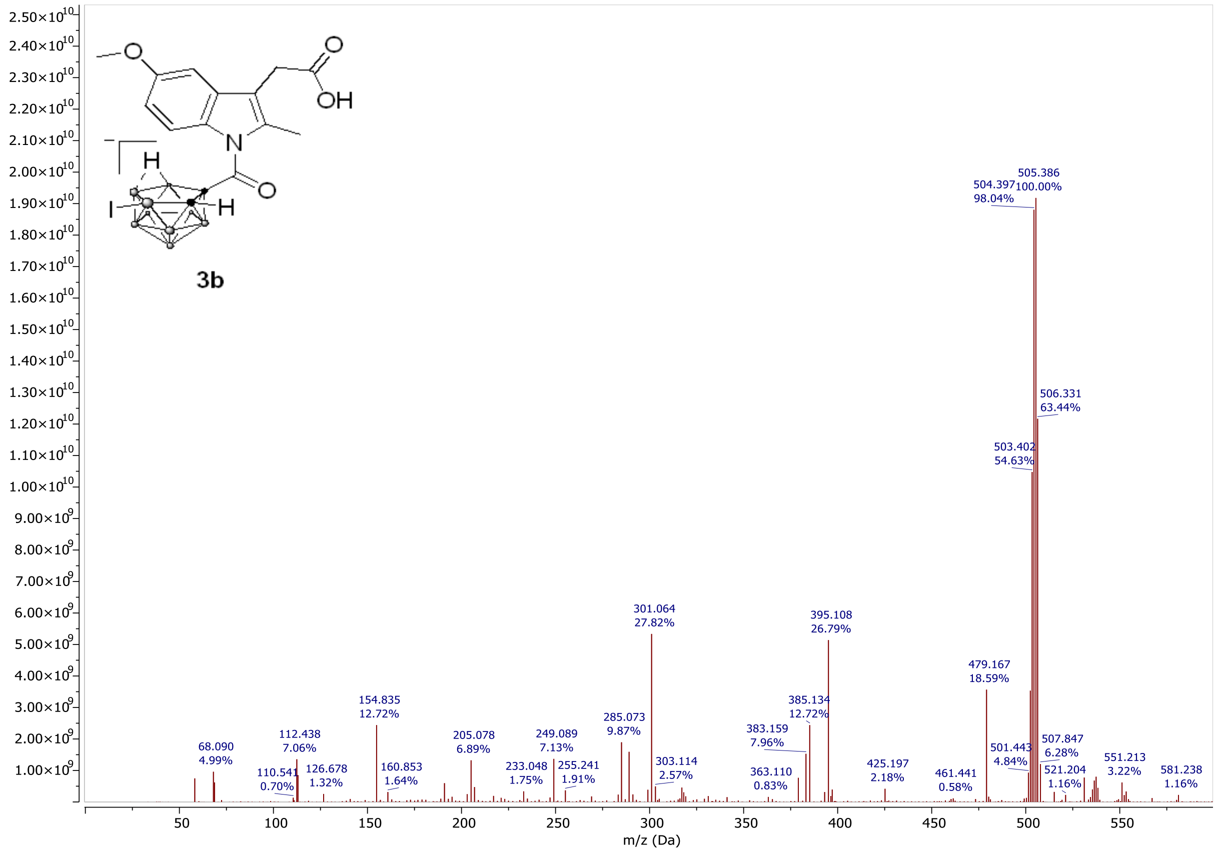

Figure S 14. m/z (MS-ESI⁻) of **3b** (system 4).

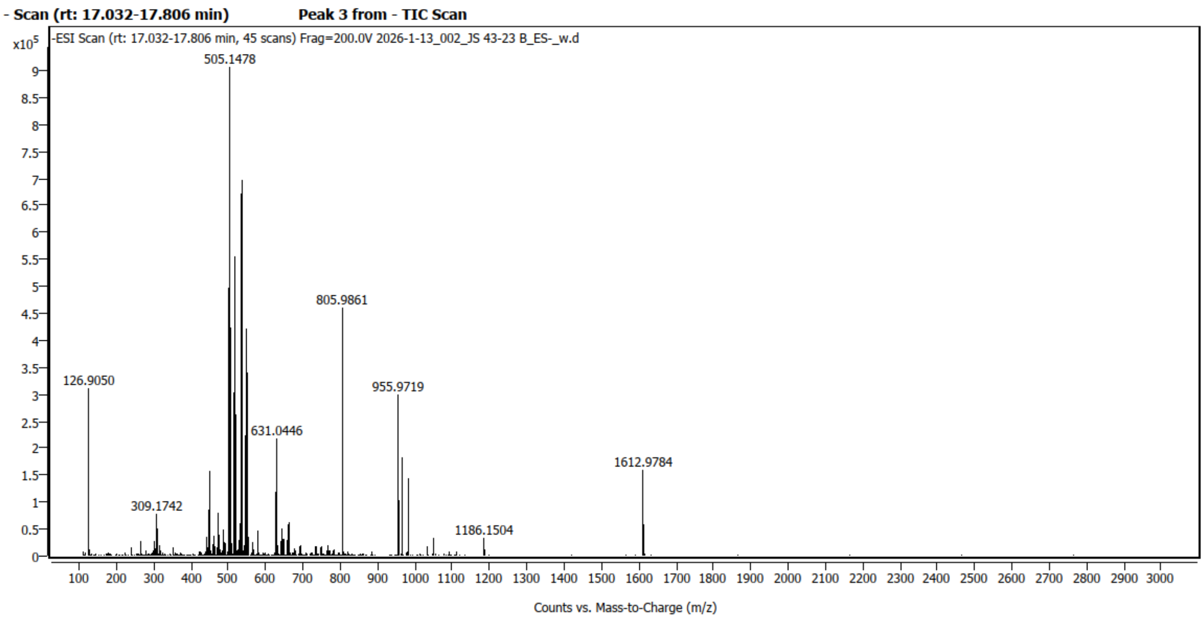

m/z	Z	Abund	Abund %
126.9050		310737	34.29
265.1480		27603	3.05
303.0720		26698	2.95
304.0683		27689	3.06
309.1742		77198	8.52
311.1689		50998	5.63
442.9843		33826	3.73
443.9807		34381	3.79
448.1477		31498	3.48
449.1442		84901	9.37
450.1406		150732	16.64
451.1371	1	156365	17.26
452.1339	1	72822	8.04
462.1406		34334	3.79
463.1372		36078	3.98
473.1442		43530	4.80
474.1406		76813	8.48
475.1372	1	80129	8.84
476.1343	1	38779	4.28
477.1526		24871	2.74
487.1598		25901	2.86
488.1563		45376	5.01
489.1529	1	48259	5.33
490.1505	1	25924	2.86
501.1619		44115	4.87
502.1583		179562	19.82
503.1548		496152	54.76
504.1513		876085	96.69
505.1478	1	906088	100.00
506.1449	1	422432	46.62
507.1483	1	85329	9.42
508.1476		28159	3.11
515.1775		26960	2.98
516.1740		109772	12.11
517.1704		301773	33.31
518.1669	1	540959	59.70
519.1635	1	554963	61.25
519.1848	1	41129	4.54
520.1607	1	261183	28.83
520.1819	1	77612	8.57
521.1637	1	52926	5.84
521.1788	1	87282	9.63
522.1757	1	43340	4.78
531.1861		28259	3.12

m/z	Z	Abund	Abund %
532.1828		49029	5.41
533.1823		59715	6.59
534.1620		32696	3.61
534.1841		146152	16.13
535.1582		37010	4.08
535.1810		385030	42.49
536.1775		671356	74.09
537.1741	1	696658	76.89
538.1714	1	330973	36.53
539.1743	1	62865	6.94
546.1845		81483	8.99
547.1810	1	223977	24.72
548.1775	1	401871	44.35
548.1995	1	63640	7.02
549.1742	1	420473	46.41
549.1963	1	172806	19.07
550.1717	1	203736	22.49
550.1930	1	321497	35.48
551.1749	1	41002	4.53
551.1896	1	339561	37.48
552.1861	1	165803	18.30
553.1691	1	69868	7.71
553.1895	1	33354	3.68
554.1664	1	35310	3.90
567.1848		24309	2.68
580.9638		46367	5.12
628.0550		43255	4.77
629.0515		118905	13.12
630.0480		208574	23.02
631.0446	1	217618	24.02
632.0419	1	108267	11.95
643.0671		27127	2.99
644.0637		47556	5.25
645.0604	1	50540	5.58
646.0587	1	27502	3.04
648.0585		30751	3.39
649.0552		31774	3.51
660.0587		28777	3.18
661.0553		30555	3.37
661.0775		33449	3.69
662.0741		58511	6.46
663.0706	1	62367	6.88
664.0670	1	32591	3.60

m/z	Z	Abund	Abund %
805.9861	1	459693	50.73
806.9892	1	78736	8.69
955.9719	1	298820	32.98
956.9751	1	62754	6.93
957.9695	1	103763	11.45
966.0007	1	182242	20.11
967.0038	1	40809	4.50
982.9908	1	142827	15.76
983.9939	1	30637	3.38
1050.0219		32688	3.61
1186.1504		32626	3.60
1612.9784	1	159237	17.57
1613.9815	1	58293	6.43

Figure S 15. HRMS of **3a**.

COX Inhibition Assay

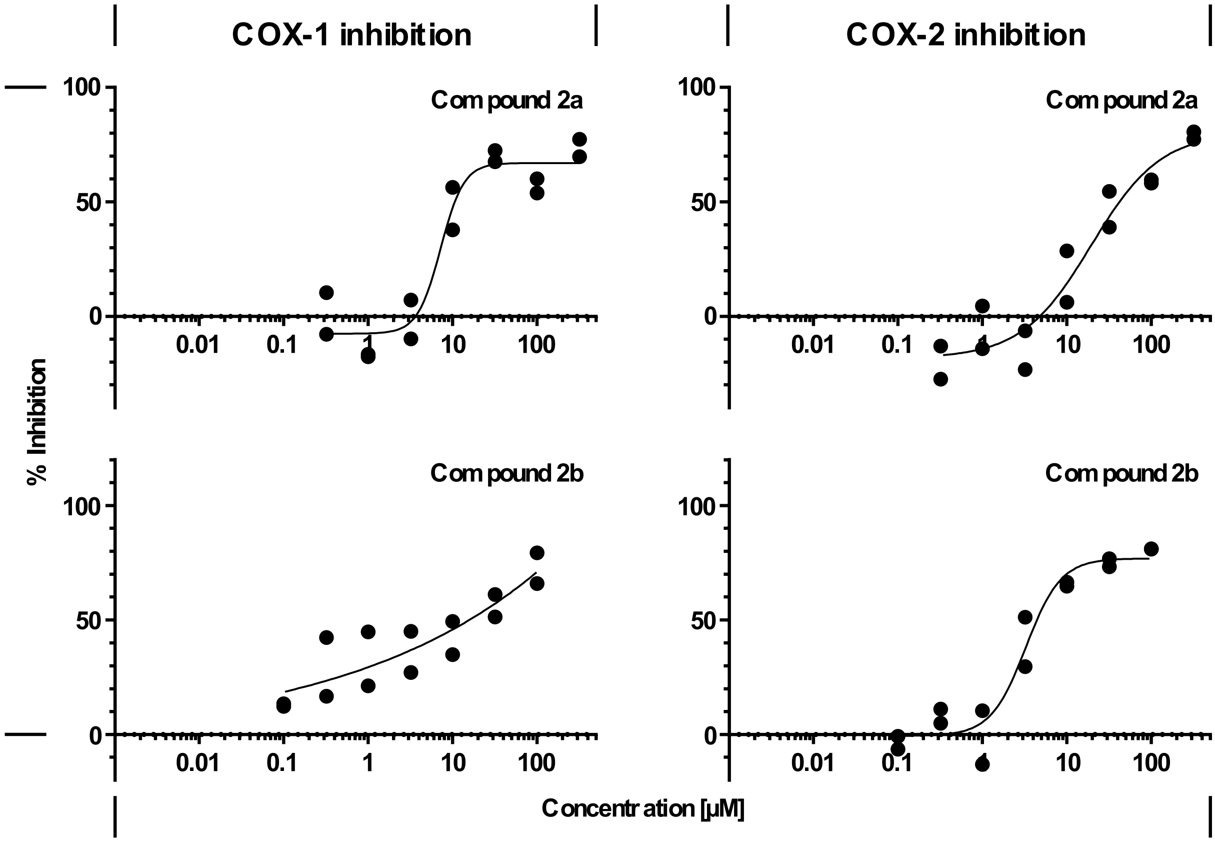

Figure S 16. COX inhibition assay. COX-1 (left) and COX-2 inhibition (right) of **2a** (top) and **2b** (bottom).

Optimization of radioiodination

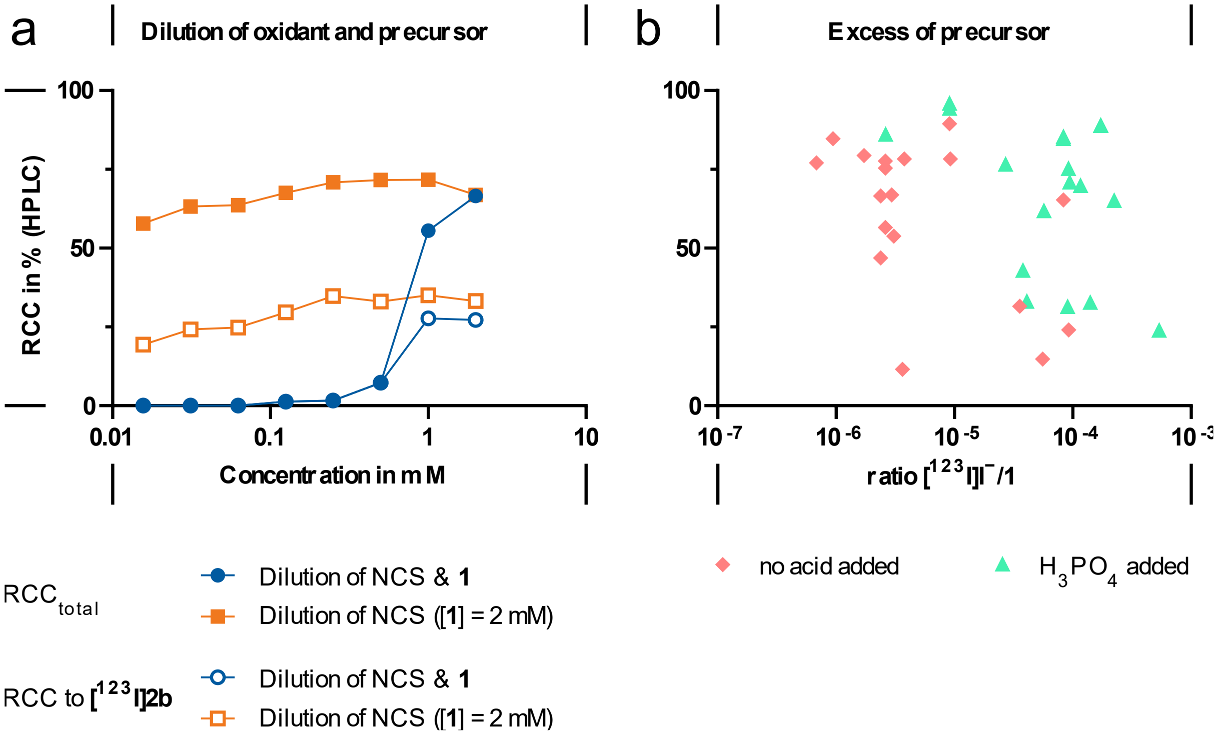

Figure S 17. Optimization of radioiodination.

a Dilution of oxidant NCS (and precursor **1**) in the absence of H_3PO_4. **b** Overview of all n.c.a. experiments conducted without acid addition and with H_3PO_4 added (10 mM final concentration, approx. pH 5), respectively . RCC dependent on precursor excess.

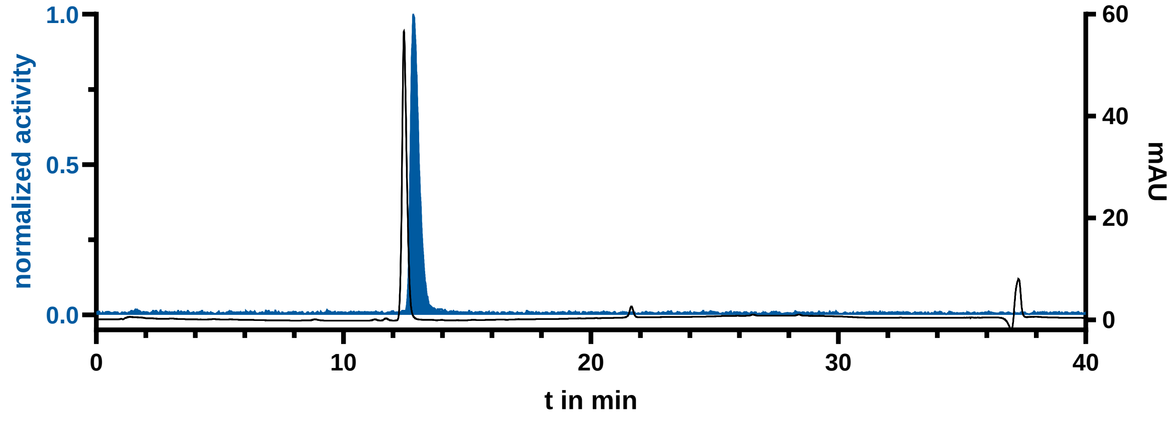

Figure S 18. **$[^{123}I]$2b** coinjected with **2b** (system 6 gradient 3).

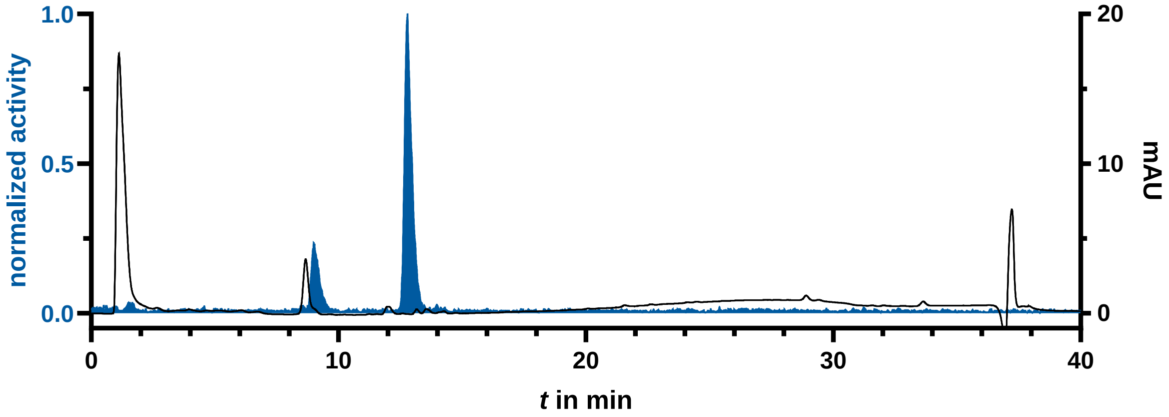

Figure S 19. Sample of **[^{123}I]2b** (t_R = 12.4 min) withdrawn after 16 h of incubation with human plasma, coinjected with **3b** (t_R = 8.7 min, system 6 gradient 3).

Murine liver microsome assay

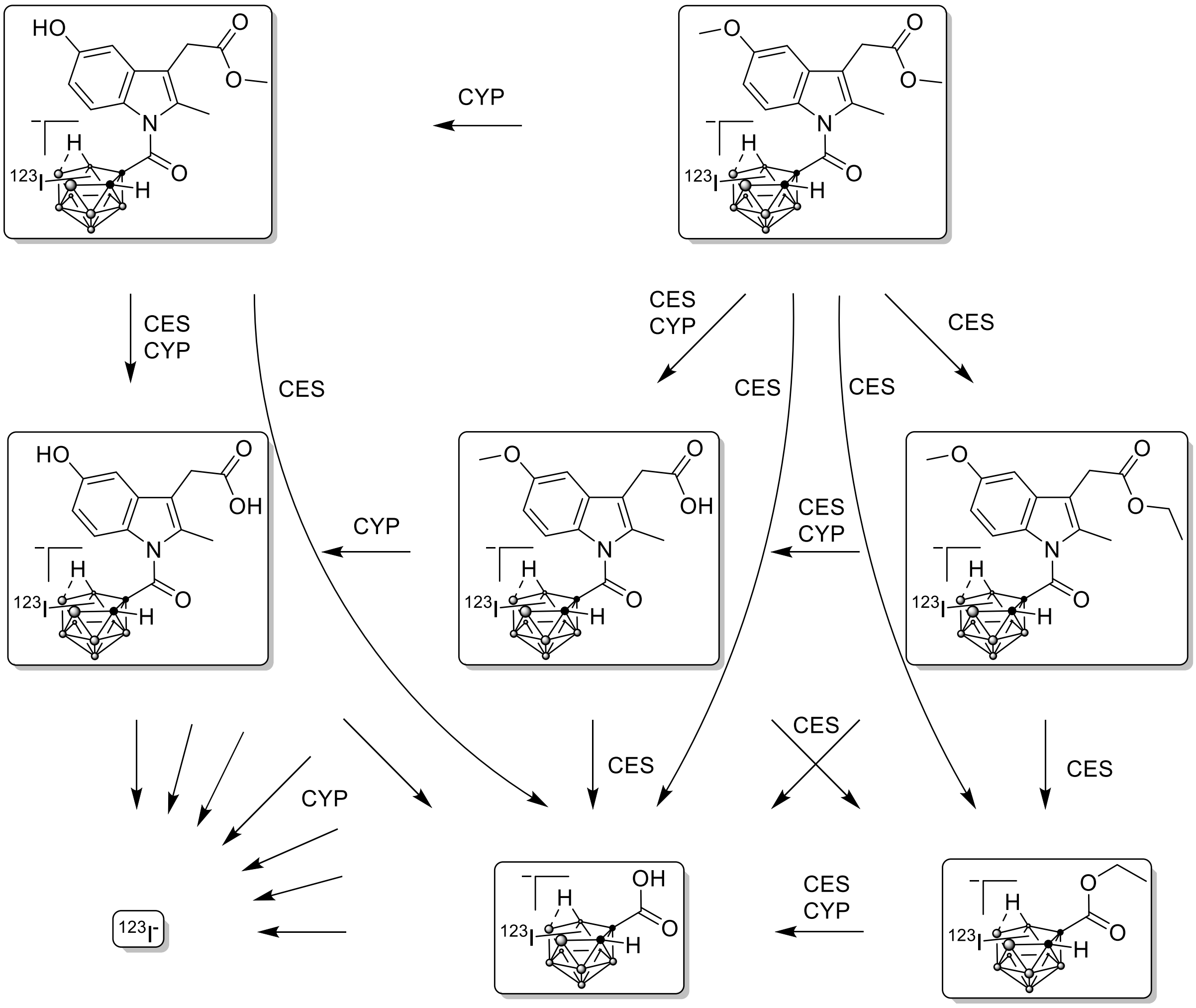

Figure S 20. Possible radiometabolites of **[^{123}I]2b** obtained in murine liver microsome assay as result of CYP and CES metabolism based on literature reported metabolism of indomethacin [64–66].

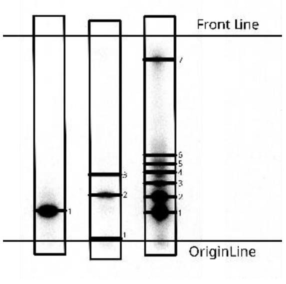

Figure S 21. Radio TLC of murine liver microsome assay. Lanes from left to right: reference **[^{123}I]2b**, reference **[^{123}I]3b**, 60 min control.

Cell uptake studies

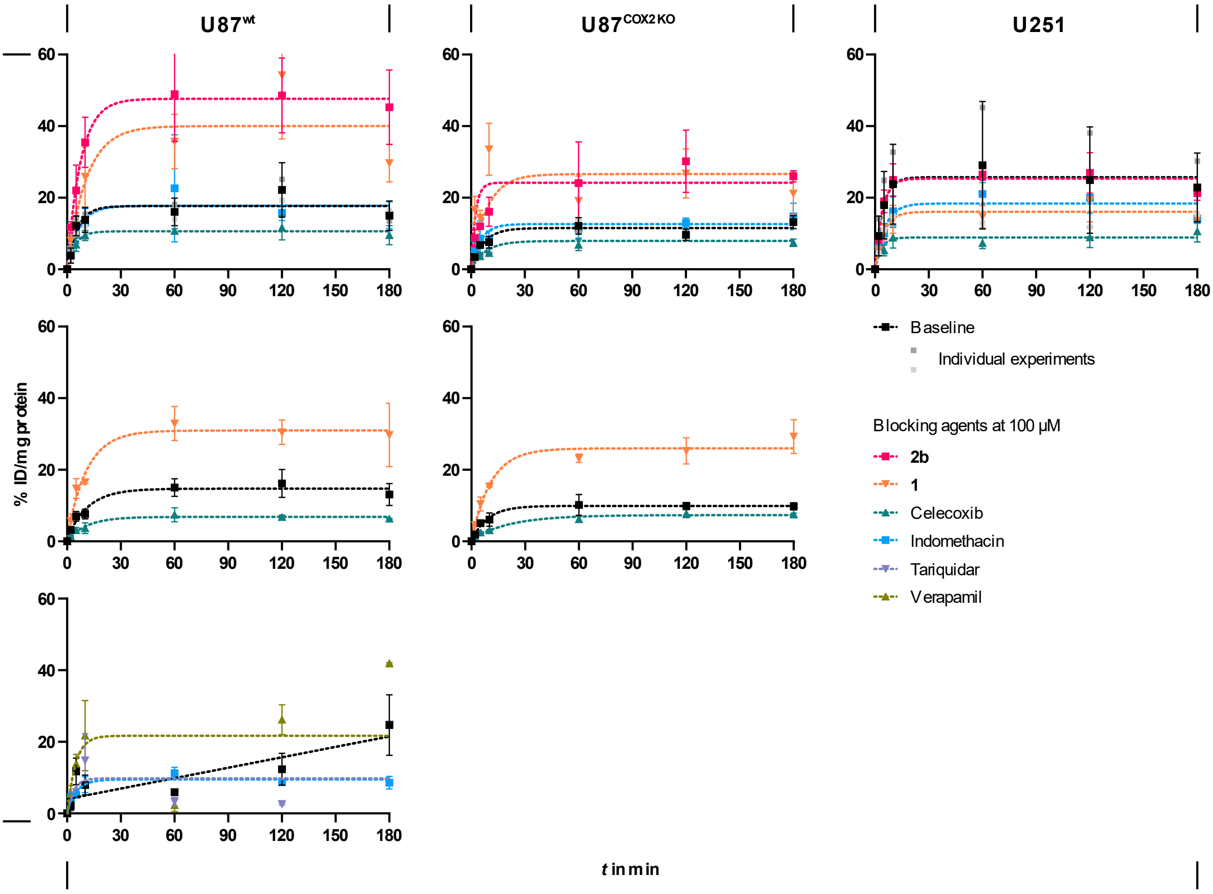

Figure S 22. Cell uptake studies of **[123I]2b** in U87 (left column), U87^{COX2KO} (middle column), and U251 cells (right column).

Each row represents a set of 2 (top row) and 1 (middle and bottom row) experiments, respectively, with cells seeded from the same passage. Uptake in % initial dose (ID)/mg protein is represented as mean ± SD of three individual values. In the top row, mean and SD are calculated from six individual values. Means of single experiments are shown as well.

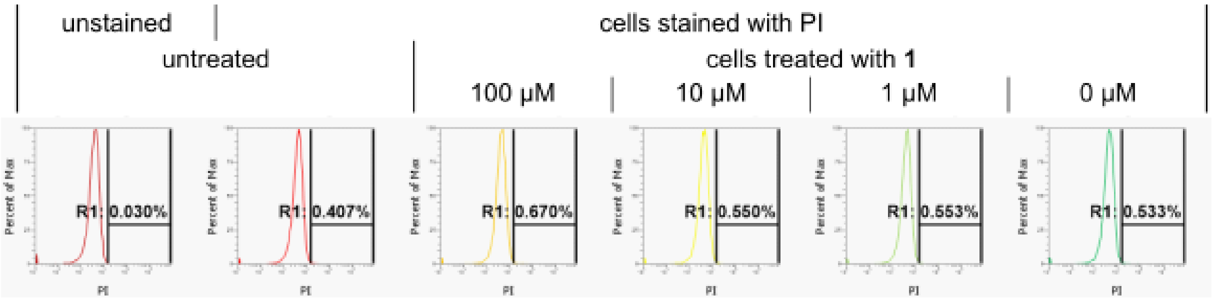

Figure S 23. Flow cytometry analysis of U87 cells treated with different concentrations of **1**.

Histograms of propidium iodide (PI) fluorescence from gated single cells are shown separately for all conditions, with the percentage of PI-positive cells indicated for each histogram.

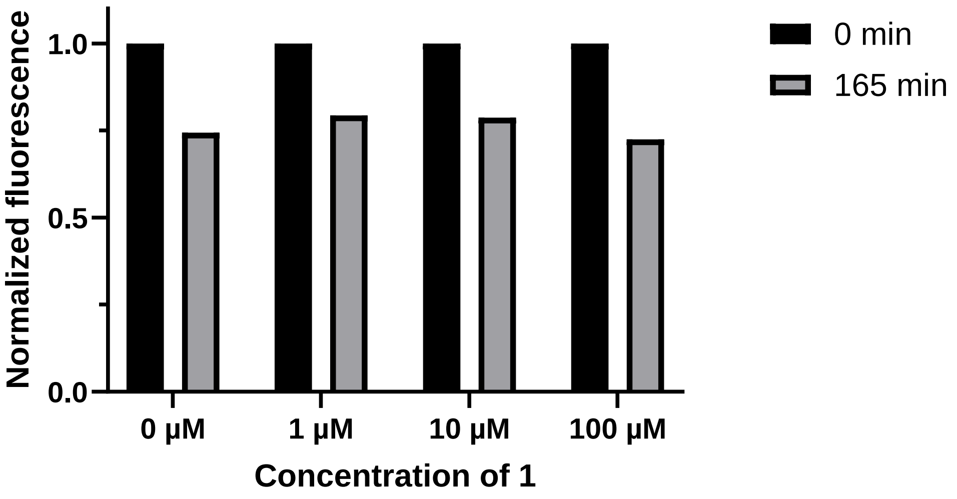

Figure S 24. Calcein efflux from U87 cells treated with different concentrations of **1**.

Calcein fluorescence (excitation 496 nm, emission 517 nm) was determined at indicated time points, corrected for unstained cells, and normalized to the initial fluorescence measured for each concentration.

Urine analysis

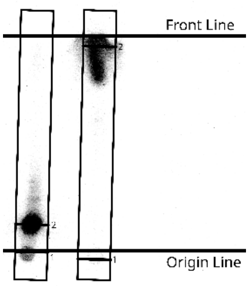

Figure S 25. Radio-TLC of urine sample collected 1.5 h after injection of **[^{123}I]2b** in U87 xenografted mouse.

Left lane: reference compound **[^{123}I]2b**, right lane: sample after protein precipitation using 15% v/v TCA.
